# Supplementary material for: The effect of using games in teaching conservation
Source: PeerJ. 2018 Apr 30;6:e4509. doi: 10.7717/peerj.4509 (PMC5936071; doi:10.7717/peerj.4509)
Supplement: Supplemental Information 1 — Figure showing relationship between overall perception score and undirected learning score. There was a significant interaction between lesson type and undirected learning score (General Linear Mixed Model models: χ2 = 7.29, df = 2, p = 0.026). The correlation coefficient for Didactic Instruction is 0.009, that for Supplemental Game is 0.005 and that for Experiential Game is 0. [file peerj-06-4509-s001.pdf]

Supplementary Figure S1

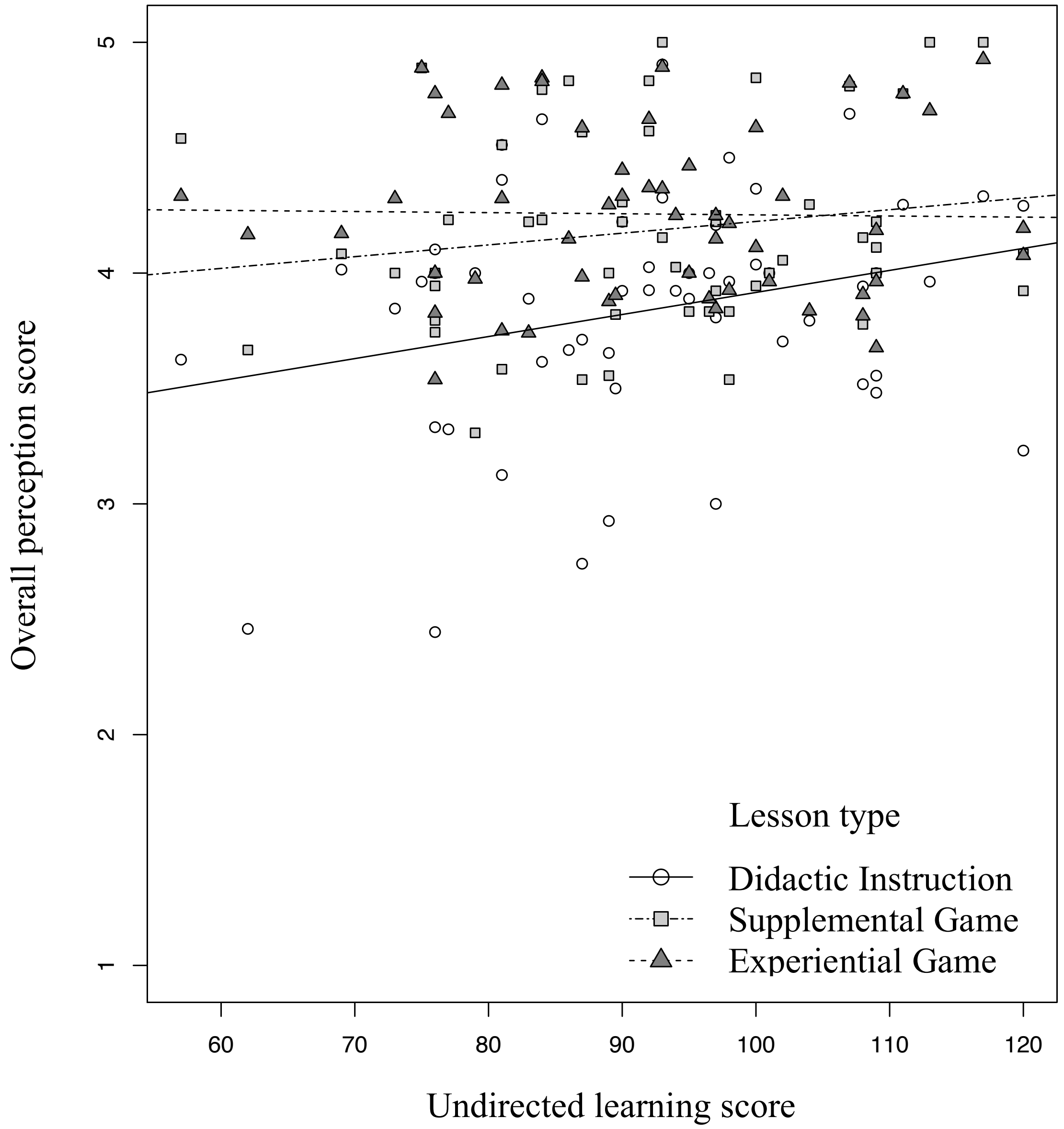

Figure S1. Figure showing relationship between overall perception score and undirected learning score. There was a significant interaction between lesson type and undirected learning score (General Linear Mixed Model models: $\chi^2 = 7.29$, df = 2, p = 0.026). The correlation coefficient for Didactic Instruction is 0.009, that for Supplemental Game is 0.005 and that for Experiential Game is 0.
