# Supplementary material for: The effect of using games in teaching conservation
Source: PeerJ. 2018 Apr 30;6:e4509. doi: 10.7717/peerj.4509 (PMC5936071; doi:10.7717/peerj.4509)
Supplement: Supplemental Information 2 — Learning as measured by proportion of quiz questions correct. Quizzes were conducted before, after or one-week post-lesson (period). Different lines denote different lesson types. (A) Reproduction-type questions; (B) Meaning-type questions; (C) Applicationtype questions. Error bars denote standard errors of sample size (nboth years = 55; nyear 2015 = 30; year 2016 = 25). When testing for a significant interaction between period and lesson type, a significant interaction was found for Reproduction-type questions both years (Bi; Generalised Linear Mixed Model with Binomial error distribution: χ2 = 30.02, df = 2, p < 0.001) and for Reproduction-type questions year 2015 (Bii; Generalised Linear Mixed Model with Binomial error distribution: χ2 = 33.71, df = 2, p < 0.001). Details of results are shown in Table S5. DI–Didactic Instruction; SG–Supplemental Game; EG–Experiential Game. [file peerj-06-4509-s002.pdf]

Supplementary Figure S2

A i. Both years

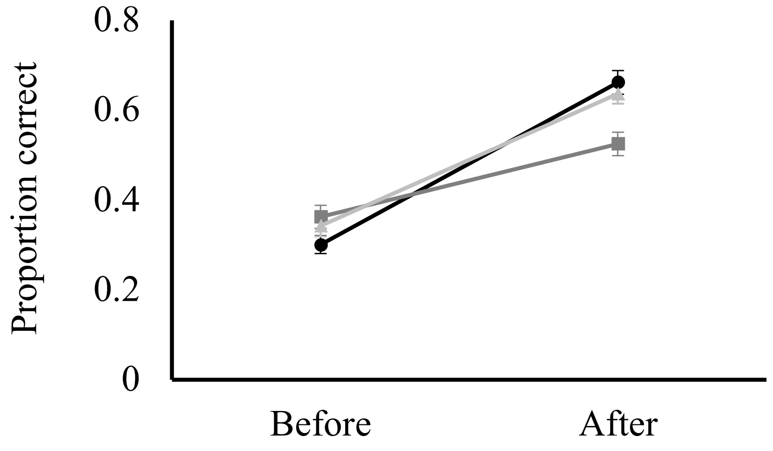

ii. Year 2015

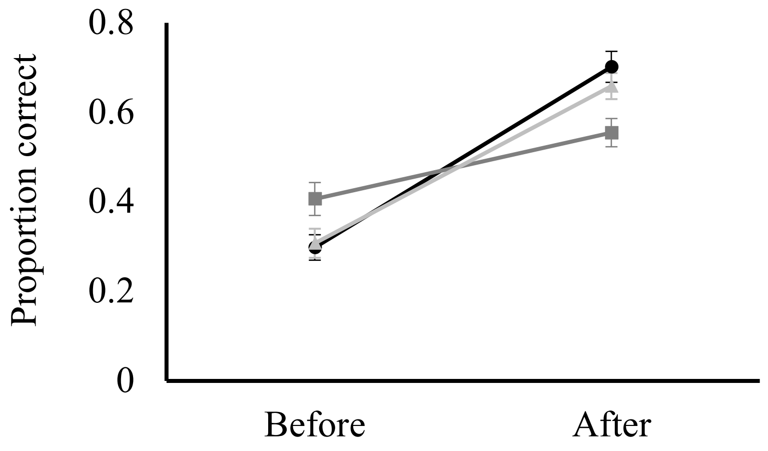

iii. Year 2016

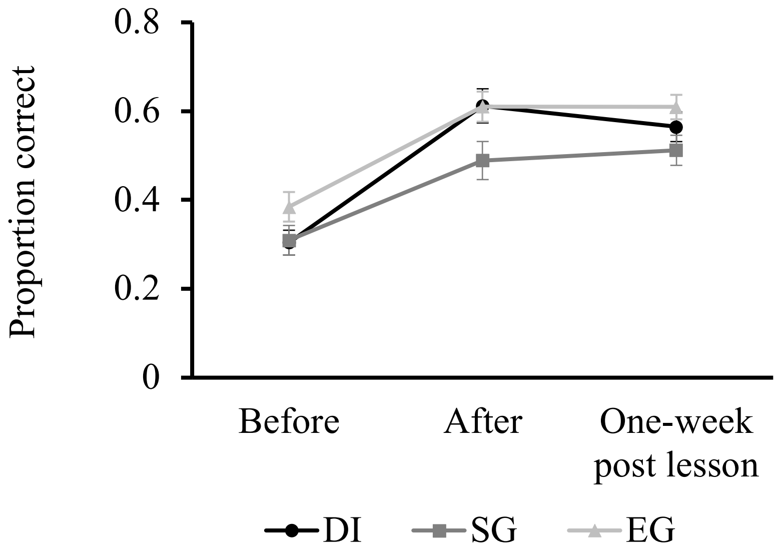

B i. Both years

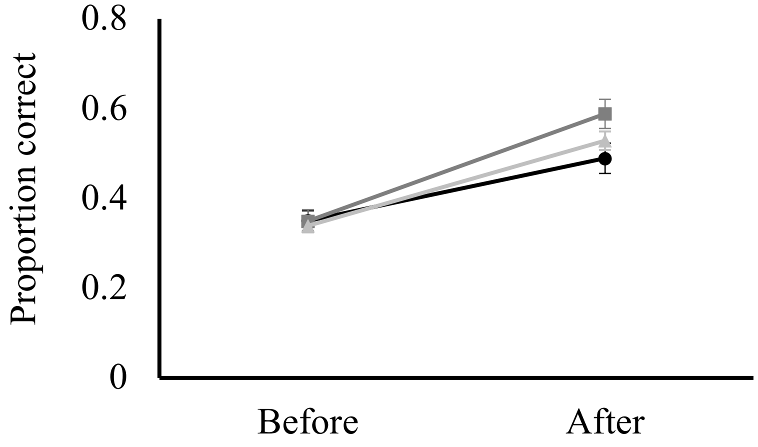

ii. Year 2015

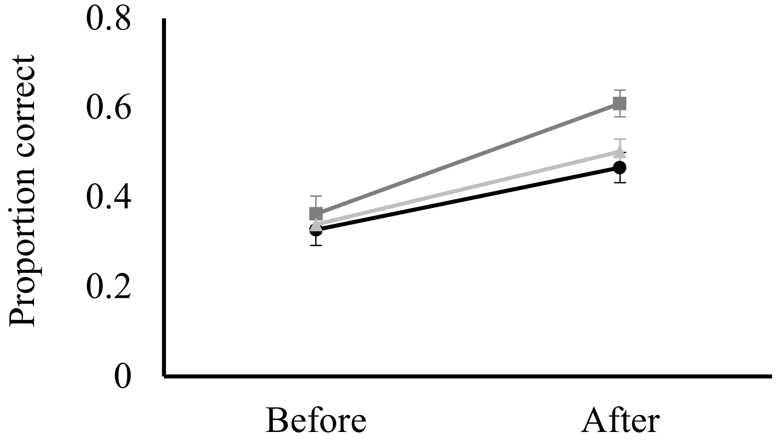

iii. Year 2016

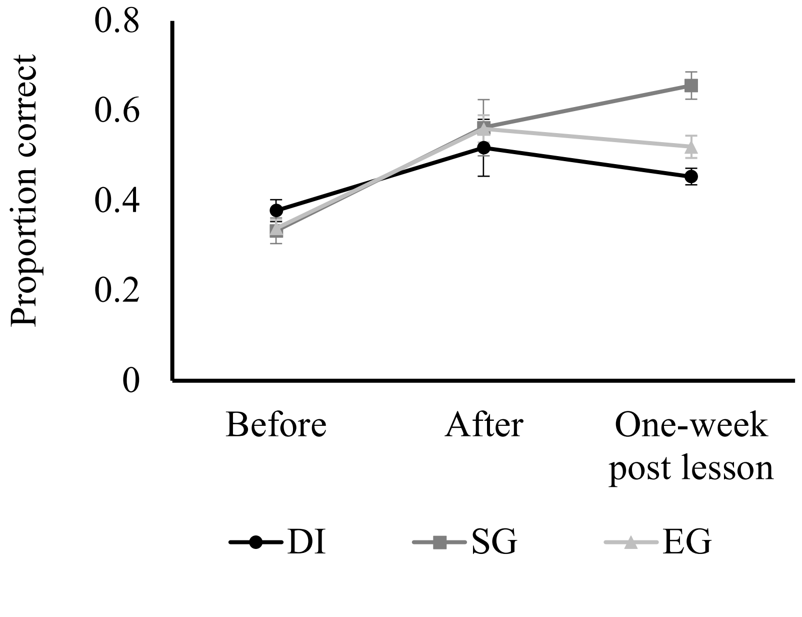

C i. Both years

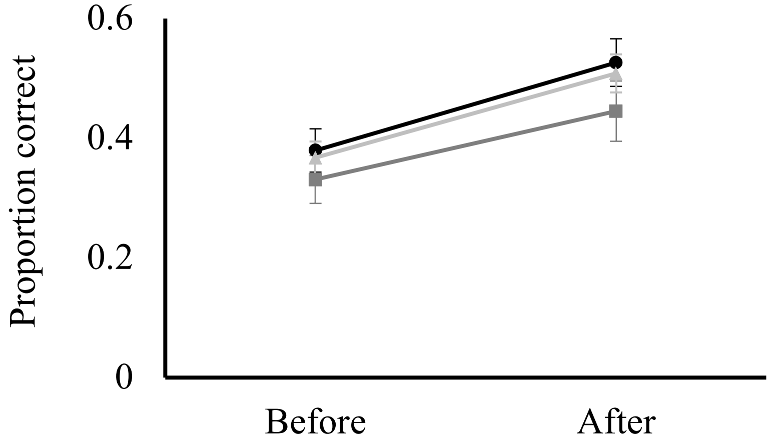

ii. Year 2015

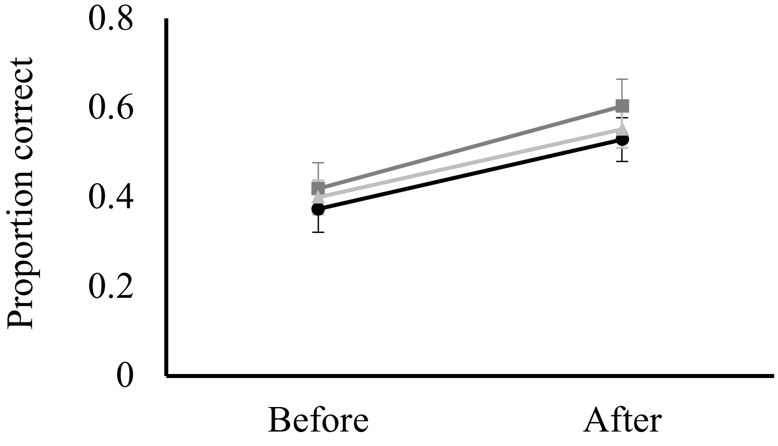

iii. Year 2016

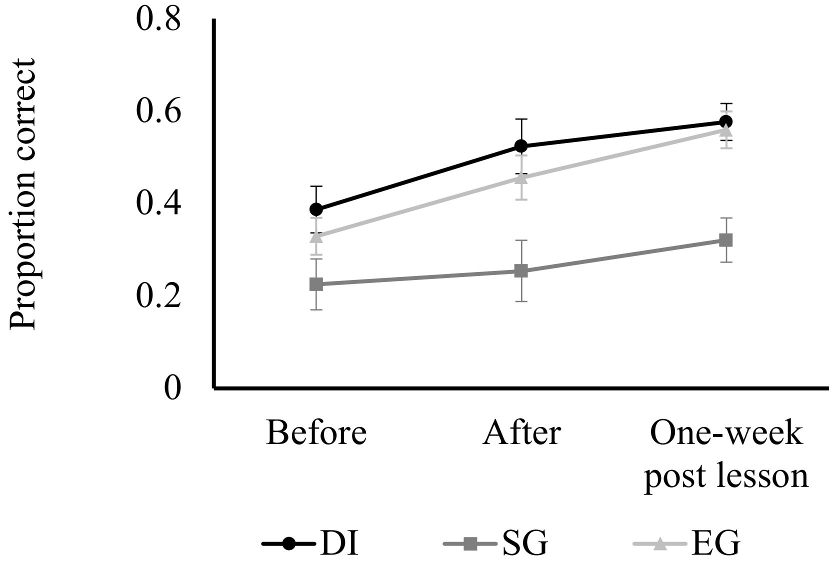

Figure S2. Learning as measured by proportion of quiz questions correct. Quizzes were conducted before, after or one-week post-lesson (period). Different lines denote different lesson types. (A) Reproduction-type questions; (B) Meaning-type questions; (C) Application-type questions. Error bars denote standard errors of sample size ($n_{both\ years} = 55$; $n_{year\ 2015} = 30$; year 2016 = 25). When testing for a significant interaction between period and lesson type, a significant interaction was found for Reproduction-type questions both years (Bi; Generalised Linear Mixed Model with Binomial error distribution: $\chi^2 = 30.02$, df = 2, $p < 0.001$) and for Reproduction-type questions year 2015 (Bii; Generalised Linear Mixed Model with Binomial error distribution: $\chi^2 = 33.71$, df = 2, $p < 0.001$). Details of results are shown in Table S5. DI – Didactic Instruction; SG – Supplemental Game; EG – Experiential Game.
