# Supplementary material for: The effect of using games in teaching conservation
Source: PeerJ. 2018 Apr 30;6:e4509. doi: 10.7717/peerj.4509 (PMC5936071; doi:10.7717/peerj.4509)
Supplement: Supplemental Information 3 [file peerj-06-4509-s003.pdf]

Section 1: Teaching Feedback Survey

Your code:

Hello! We are exploring the effectiveness of different teaching methods and would appreciate your feedback on this lesson as it will contribute to improving the delivery method.

Please tick one of the squares for each statement below.

1. **Compared to traditional teaching methods** (i.e. a previously encountered typical lesson), rate this particular lesson in terms of the

	Much Less	Slightly less	No difference	Slightly more	Much more
a. Amount of content taught	☐	☐	☐	☐	☐
b. Level of understanding of the topic	☐	☐	☐	☐	☐
c. Level of motivation for me to learn more after the lesson	☐	☐	☐	☐	☐
d. Degree of remembrance of content	☐	☐	☐	☐	☐
e. Degree in broadening my perspective on related topics	☐	☐	☐	☐	☐
f. Degree of encouragement to ask questions	☐	☐	☐	☐	☐
g. Amount of my attention retained for the length of the tutorial	☐	☐	☐	☐	☐
h. Level of engagement with the tutor	☐	☐	☐	☐	☐
i. Level of engagement with other students	☐	☐	☐	☐	☐
j. Degree of learning from my peers	☐	☐	☐	☐	☐
k. Degree of connection with my peers	☐	☐	☐	☐	☐
l. Level of challenge	☐	☐	☐	☐	☐
m. Degree of nurturing creative thinking	☐	☐	☐	☐	☐
n. Degree of appreciation of the application of this topic	☐	☐	☐	☐	☐

Section 2: Intrinsic Motivation

3. For each of the following statements, please indicate how true it is for you, using the following scale:

1	2	3	4	5	6	7
not at all true		somewhat true				very true

a. While I was working on the lesson I was thinking about how much I enjoyed it. _____

b. I did not feel at all nervous about doing the lesson. _____

c. I felt that it was my choice to do the lesson. _____

d. I think I am pretty good at this lesson. _____

e. I found the lesson very interesting. _____

f. I felt tense while doing the lesson. _____

g. I think I did pretty well at this activity, compared to other students. _____

h. Doing the lesson was fun. _____

i. I felt relaxed while doing the lesson. _____

j. I enjoyed doing the lesson very much. _____

k. I didn't really have a choice about doing the lesson. _____

l. I am satisfied with my performance at this lesson. _____

m. I was anxious while doing the lesson. _____

n. I thought the lesson was very boring. _____

o. I felt like I was doing what I wanted to do while I was working on the task. _____

p. I felt pretty skilled at this lesson. _____

r. I felt pressured while doing the lesson. _____

s. I felt like I had to do the lesson. _____

u. I did the lesson because I had no choice. _____

v. After working at this lesson for awhile, I felt pretty competent. _____

Do you have any **teaching experience:** yes, no
Do you have any **formal training in teaching:** yes, no

Section 3: Bondedness Survey

Your code:

Hello! We are exploring the effectiveness of different teaching methods on social bonding and would appreciate your feedback on this lesson as it will contribute to improving the delivery method.

In the table below, enter the name of each group member and indicate a number 1-7 that best describes your relationship to the other members in your group before and after the lesson.

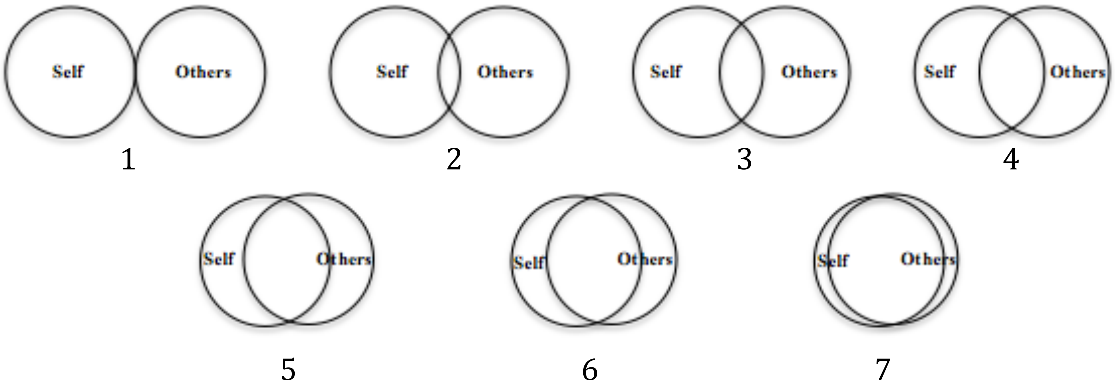

**Group member name**	**Before lesson relationship**	**After lesson relationship**	**How well do you know this group member?** (1 - not at all, 7 very much so)
