# Supplementary material for: The effect of using games in teaching conservation
Source: PeerJ. 2018 Apr 30;6:e4509. doi: 10.7717/peerj.4509 (PMC5936071; doi:10.7717/peerj.4509)
Supplement: Supplemental Information 4 [file peerj-06-4509-s004.docx]

Supplementary Methods S1

***Personality and learning style questionnaires***

The big five personality questionnaire consisted of 70 statements, 14 for each of the component: extraversion, agreeableness, conscientiousness, neuroticism and openness to experience (Elshout & Akkerman 1975). An example of a statement is “I am the life of the party.'' and one has to indicate on a five-point scale how well this description fits him or her. Agreeable persons are cooperative as well as likeable, and have an optimistic view of human nature. Conscientiousness relates to an individual’s tendency to be organized and self-disciplined. Extroverts are sociable and often thought of as full of energy. Neuroticism refers to the lack of positive psychological adjustment and the tendency to exhibit negative emotions. Here, a low score means more neurotic. Openness to experience is characterized by intellectual curiosity and creativity.

The Inventory of Learning Styles (ILS) questionnaire comprises of 120 statements categorised into 20 subscales, measuring different aspects of processing strategies, regulation strategies, learning orientations and mental models of learning (Vermunt 1994). For each statement, a student has to indicate on a five-point scale to what extent it is reflective of his or her learning style. Students with a reproduction-directed learning style are mainly focused at memorising, rehearsing and reproducing what is learnt; learning is viewed as the intake of knowledge. Students with a meaning-directed learning style typically seek to understand what is meant exactly in their study material, interrelate subject matters and strive critically to develop their personal view. Students with an application-directed learning style attempt to use the knowledge on real-world settings. Finally, students with an undirected learning style are typified by the lack of regulation and emphasis is placed on cooperating with fellow students and on stimulating education. With the ILS, it is possible to quantify each of the four styles in a summated score. Students, therefore, show characteristics of each style but, as Vermunt assumes, one style dominates. The ILS is being applied on a large scale in higher education in Holland.

***Game design and teaching methods***

Based on recommended guidelines for creating educational games (Linehan et al. 2011), games (SG and EG) were designed with the following principles in mind:

- provide a clear goal
- allow multiple paths to reach end-goal
- provide feedback on progress

Students were introduced to the gaming objective at the beginning of the lesson. A typical gaming session comprises a repeated three-step process: students were first introduced to the concepts of a topic as in didactic instruction, then given a question and subsequently allowed a move in the game if the question was answered accurately. Therefore, the process incorporates elements of didactic instruction in which knowledge was delivered, as well as gaming in which strategic moves were made. When a question was posed on a slide projected in view of the class, all teams had to answer the question, either by writing on a small whiteboard or choosing an option for a multiple-choice question. In most games, answers were hidden from other teams either by covering the whiteboards or by using a game cover. Answers could be seen in a few games, for e.g. in the conservation genetics casino, bets on multiple choice answers were placed on a common board. After a time limit, all teams would present their answers to the class and the tutor would be involved in the discussion and was able to provide feedback.

Both SG and EG share elements of game design mentioned above. The difference lies in whether the game had any meaningful link with the subject matter. In SG, the game had no meaningful relationship with the topic, the goal was either to get to the end of a race or to obtain the most number of points. However, in EG, students did tasks related to the subject matter, the goal was similar to a topic-related achievement in the real world. For e.g. in the ‘Behavioural Ecology’ topic, teams were to behave as different species in order to eat or be eaten by other teams (Table 1). Further, EG required participants to take actions or decisions to succeed. Game moves were associated with question and were flexible, giving teams control of their actions towards different paths to success. For example, a multiple-choice question in conservation genetics would allow teams to switch the colour of their chips and an open-ended question would allow teams to double their chips if answers were correct. Teams can therefore decide how many chips and which coloured chip to bet. We also incorporated random events or situations in EG to reflect stochasticity prevalent in biology. For example, a stochastic forest fire event eliminated 50% of the casino chips of all teams in conservation genetics, or an elephant killed one of the villagers in the human-wildlife conflict lesson and the teams have to decide on a course of action. Therefore, EG closely reflected reality.

***Assessment***

In section 2 of the perception questionnaire, students completed a shortened version of the intrinsic motivation inventory ((Ryan et al. 1991); Supplementary File (Questionnaire)), which contains 20 items such as “While I was working on the lesson I was thinking about how much I enjoyed it.” and “I felt that it was my choice to do the task.” Items were rated on a 7- point Likert scale from 1 (strongly disagree) to 7 (strongly agree). The 20 items covered 4 subscales. The interest/enjoyment subscale is the self-report measure of intrinsic motivation. The perceived choice and perceived competence subscales are positive predictors of both self-report and behavioral measures of intrinsic motivation, and pressure/tension is a negative predictor of intrinsic motivation. The third section was aimed at measuring interpersonal interactions between students within teams, which is a positive predictor of intrinsic motivation. Students answered questions that have previously been used as indicators of social bonding (Wiltermuth & Heath 2009; Davis et al. 2015). The first question was based on the Inclusion of Other in Self (IOS) Scale (Aron et al. 1992): “Please circle the picture that best describes your relationship to the other participants in your group” (Supplementary File (Questionnaire)). Students also answered the following question in order to control for potential confounds: “How much do you know [each of] the other participants?”.

***Statistical analysis***

All analyses were conducted using R 3.0.2. For the first section of the questionnaire, we examined the effects of lesson type (DI, SG or EG) on students’ perceptions using a ordinal logistic model (ordinal package; (Christensen 2010)) with level of agreement as the response, lesson type as the fixed factor, and student and topic as random factors. Year, course (Wildlife Conservation Course or Diploma), age, gender, teaching experience (yes or no), formal teaching training (yes or no) were included as covariates. The probit link function was used to account for heteroskedasticity. We used one model for each statement. When the proportional odds assumption of ordinal logistic models was violated, we used the partial proportional odds (PPO) model with a logistic link function instead (e.g. Sasidharan & Menéndez 2014). The random effects were omitted in these (PPO) models to improve simplicity and to achieve convergence. Post-hoc tests on the ordinal logistic models were conducted to examine the differences in responses among the three lesson types. Since we were unable to conduct post-hoc tests on PPO models, we used ordinal logistic models for the post-hoc tests.

To examine if students’ perception of the lessons varied with each component of learning style, we first averaged questionnaire scores across all statements of the first section for each student for each lesson. This average questionnaire score was entered as the response. We then used separate General Linear Mixed Model models with each component of learning style score (e.g. Application-directed learning style) and lesson type and their interaction as fixed factors. Random factors and covariates entered were the same as above. Similarly, to examine if students’ perception differed with each component of the big five personality, we used separate General Linear Mixed Model models with each factor of personality trait (e.g. conscientiousness score) and lesson type and their interaction as fixed factors. Models fulfilled homogeneity of variance and normality of residuals assumptions.

We analysed variation in intrinsic motivation and its parameters using a similar approach. First, we calculate the intrinsic motivation, perceived choice, perceived competence and pressure/tension scores for each student for each lesson. These scores were the response variables. We then used separate ordinal logistic models for each response with lesson type as the fixed factor, and student and topic as random factors. Year, course, age, gender, teaching experience, formal teaching training were included as covariates.

Variation in bondednesss was analysed using ordinal logistic model with after bondedness score minus that of before as the response variable. We included the rating student, the rated student and topic as random factors. Age and gender of rating student, as well as the level of ‘how well you know’ the rated person were entered as covariates.

We analysed the variation in each behaviour (joyful, distracted, question-asking and question-answering) with two analyses: (i) a Generalised Linear Mixed Model (GLMM) with a binomial error distribution, the ‘probability of performing the behaviour’ as a response variable, and (ii) a Linear Mixed Model (LMM) with a normal error distribution and the transformed ‘rate of behaviour per hour’ as the response variable, omitting zero counts (i.e. .where students did not perform behaviour; (Bates et al. 2014). Data for ‘rate of behaviour’ attained normality of residuals and homogeneity of variance after transformation. In checking whether our distribution of ‘rate of behaviour per hour’ fulfilled the normality assumption, we found no outliers, suggesting that we had no one/two students who were asking most of the questions. We omitted analysing the effect of lesson type on rate of question-answering as in almost all game lessons, all students were to answer the question by writing on whiteboards and so question-answering (in the vocal sense) behaviour was minimal. Student and topic were entered as random factors. Year, course, age, gender, teaching experience, formal teaching training were included as covariates. Fixed factors included the students’ extroversion scores, lesson type and the interaction between these two. The significance of the fixed factors was assessed using the likelihood-ratio test on models with and without the fixed factor (Bates et al. 2014). The GLMM on ‘probability of performing a distracted behavior’ did not converge due to the a relatively fewer number of this behavior compared to other behaviors. The omission of ‘Year’ as a covariate in that model resolved this issue.

To examine whether learning effectiveness differed with lesson type for the three topics, we entered in a GLMM with binomial error distribution, correctness as a response (1 = correct; 0 = wrong), lesson type and period (before or after or long-term post lesson (for 2016 only)) and their interaction as fixed factors, and topic and student as random variables. We did one analysis for both years combined with period having only two levels (before or after lesson), one analysis for year 2015 (period: before or after lesson) and one analysis for year 2016 (period: before or after or long-term post lesson). Further, we separated questions by question type (reproduction-, meaning or application-type) and analysed them as different response variables. In these models, we added as a covariate the corresponding component of learning style as a covariate, for e.g. reproduction-directed learning score for the model analysing variation in reproduction-type question. In all, we had 12 models, 3 for the different year groupings (both years, 2015 or 2016) in combination with 4 for the different question types (all question types; reproduction-, meaning or application-directed). We found no overdispersion (dispersion parameter >1.5) or underdispersion (<0.5) in any of our Generalised Linear Mixed Models.

**Literature cited**

Aron A, Aron EN, Smollan D. 1992. Inclusion of Other in the Self Scale and the structure of interpersonal closeness. Journal of personality and social psychology **63**:596.

Bates D, Maechler M, Bolker B, Walker S. 2014. lme4: Linear mixed-effects models using Eigen and S4. R package version **1**.

Christensen RHB. 2010. ordinal—regression models for ordinal data. R package version **22**.

Davis A, Taylor J, Cohen E. 2015. Social Bonds and Exercise: Evidence for a Reciprocal Relationship. PLOS ONE **10**:e0136705.

Elshout JJ, Akkerman AE 1975. Vijf persoonlijkheidsfaktoren test 5PFT: Handleiding [The Five Personality Factor Test (5PFT): Manual]. Berkhout B.V., Nijmegen, the Netherlands.

Linehan C, Kirman B, Lawson S, Chan GG. 2011. Practical, appropriate, empirically-validated guidelines for designing educational games. Pages 1979-1988. Proceedings of the SIGCHI Conference on Human Factors in Computing Systems.

Ryan RM, Koestner R, Deci EL. 1991. Ego-involved persistence: When free-choice behavior is not intrinsically motivated. Motivation and Emotion **15**:185-205.

Sasidharan L, Menéndez M. 2014. Partial proportional odds model—An alternate choice for analyzing pedestrian crash injury severities. Accident Analysis & Prevention **72**:330-340.

Vermunt JDHM. 1994. Inventory of learning styles in higher education; scoring key for the inventory of learning styles in higher education.

Wiltermuth SS, Heath C. 2009. Synchrony and Cooperation. Psychological Science **20**:1-5.
