# Supplementary material for: The effect of using games in teaching conservation
Source: PeerJ. 2018 Apr 30;6:e4509. doi: 10.7717/peerj.4509 (PMC5936071; doi:10.7717/peerj.4509)
Supplement: Supplemental Information 5 — Table of perception results as analysed using ordinal logistic models. Where the proportional odds assumption was violated, the partial proportional odds model was used instead. This was the case for the following statements: ‘Level of motivation for me to learn more after the lesson,’ ‘Degree of nurturing creative thinking,’ ‘Degree of encouragement to ask questions’) and in these models, random factors had to be omitted and hence the random factor variances could not be estimated. DI–Didactic Instruction; SG–Supplemental Game; EG–Experiential Game. Definition of other explanatory variables [reference level for discrete variables]: Year–year of course [2015]; Course–Diploma in Oxford or Wildlife Conservation Course in Malaysia [Diploma]; Age–age of student as an ordinal variable; Gender–gender of student [Female]; Teaching experience–whether student had teaching experience [No]; Formal training in training–whether student had any formal training in training [No]; Topic–topic of lesson. The second column shows either (i) the estimate of the slope for a continuous fixed variable, (ii) the estimate of the difference in mean from the reference level for a discrete fixed variable, or (iii) the variance of a random variable. Significant variables are highlighted in bold. For overall rating, the estimate for the DI slope is shown and the estimate for SG and EG are the respective differences in slope from that of DI. [file peerj-06-4509-s005.docx]

Supplementary Table S1. Table of perception results as analysed using ordinal logistic models. Where the proportional odds assumption was violated, the partial proportional odds model was used instead. This was the case for the following statements: ‘Level of motivation for me to learn more after the lesson’, ‘Degree of nurturing creative thinking’, ‘Degree of encouragement to ask questions’) and in these models, random factors had to be omitted and hence the random factor variances could not be estimated. DI – Didactic Instruction; SG – Supplemental Game; EG – Experiential Game. Definition of other explanatory variables [reference level for discrete variables]: Year – year of course [2015]; Course – Diploma in Oxford or Wildlife Conservation Course in Malaysia [Diploma]; Age – age of student as an ordinal variable; Gender – gender of student [Female]; Teaching experience – whether student had teaching experience [No]; Formal training in training – whether student had any formal training in training [No]; Topic – topic of lesson. The second column shows either (i) the estimate of the slope for a continuous fixed variable, (ii) the estimate of the difference in mean from the reference level for a discrete fixed variable, or (iii) the variance of a random variable. Significant variables are highlighted in bold. For overall rating, the estimate for the DI slope is shown and the estimate for SG and EG are the respective differences in slope from that of DI.

|  | Estimate (fixed)/ Variance (random) | SE | Df | Chisq | Pr(>Chi) |
| --- | --- | --- | --- | --- | --- |
| Knowledge acquisition | | | | | |
| Amount of content taught |  |  |  |  |  |
| Lesson type (SG) | 0.179 | 0.182 | 2 | 4.08 | 0.130 |
| Lesson type (EG) | 0.261 | 0.133 |  |  |  |
| Year | 0.366 | 0.247 | 1 | 2.17 | 0.141 |
| **Course** | **0.585** | **0.265** | **1** | **4.70** | **0.030** |
| Age | 0.025 | 0.131 | 1 | 0.04 | 0.848 |
| Gender | 0.198 | 0.269 | 1 | 0.54 | 0.462 |
| Teaching experience | 0.103 | 0.264 | 1 | 0.15 | 0.696 |
| Formal training in training | -0.070 | 0.360 | 1 | 0.04 | 0.846 |
| Student | 0.767 |  |  |  |  |
| Topic | 0.204 |  |  |  |  |
|  |  |  |  |  |  |
| Degree of remembrance of content | |  |  |  |  |
| **Lesson type (SG)** | **0.581** | **0.179** | **2** | **20.35** | **<0.001** |
| **Lesson type (EG)** | **0.511** | **0.132** |  |  |  |
| Year | 0.006 | 0.169 | 1 | 0.00 | 0.973 |
| **Course** | **0.027** | **0.010** | **1** | **7.79** | **0.005** |
| Age | 0.102 | 0.184 | 1 | 0.31 | 0.581 |
| Gender | -0.030 | 0.091 | 1 | 0.11 | 0.740 |
| Teaching experience | -0.013 | 0.179 | 1 | 0.01 | 0.942 |
| Formal training in training | 0.287 | 0.180 | 1 | 2.52 | 0.113 |
| Student | 0.421 |  |  |  |  |
| Topic | 0.236 |  |  |  |  |
|  |  |  |  |  |  |
| Level of understanding of the topic | |  |  |  |  |
| **Lesson type (SG)** | **0.466** | **0.187** | **2** | **8.36** | **0.015** |
| **Lesson type (EG)** | **0.257** | **0.131** |  |  |  |
| Year | 0.010 | 0.007 | 1 | 2.03 | 0.154 |
| Course | 0.023 | 0.201 | 1 | 0.01 | 0.908 |
| Age | 0.243 | 0.217 | 1 | 1.25 | 0.264 |
| Gender | 0.009 | 0.113 | 1 | 0.01 | 0.933 |
| Teaching experience | -0.051 | 0.214 | 1 | 0.06 | 0.811 |
| Formal training in training | 0.081 | 0.214 | 1 | 0.14 | 0.707 |
| Student | 0.557 |  |  |  |  |
| Topic | 0.250 |  |  |  |  |
|  |  |  |  |  |  |
| Degree of appreciation of application of topic | | |  |  |  |
| **Lesson type (SG)** | **0.132** | **0.227** | **2** | **7.04** | **0.030** |
| **Lesson type (EG)** | **0.437** | **0.167** |  |  |  |
| Year | 0.037 | 0.047 | 1 | 0.63 | 0.428 |
| Course | -0.155 | 0.316 | 1 | 0.24 | 0.625 |
| Age | -0.177 | 0.174 | 1 | 1.04 | 0.308 |
| Gender | 0.113 | 0.361 | 1 | 0.10 | 0.754 |
| Teaching experience | -0.104 | 0.331 | 1 | 0.10 | 0.754 |
| Formal training in training | -0.942 | 0.475 | 1 | 3.85 | 0.050 |
| Student | 0.592 |  |  |  |  |
| Topic | 0.000 |  |  |  |  |
|  |  |  |  |  |  |
| Development | | | | | |
| Level of motivation for me to learn more after the lesson | | | |  |  |
| **Lesson type (SG)** | **0.723** | **0.253** | **2** | **6.91** | **0.032** |
| **Lesson type (EG)** | **0.770** | **0.201** |  |  |  |
| **Year** | **0.462** | **0.191** | **1** | **5.74** | **0.017** |
| Course | 0.347 | 0.200 | 1 | 3.34 | 0.068 |
| Age | 0.114 | 0.102 | 1 | 1.21 | 0.271 |
| **Gender** | **0.564** | **0.207** | **1** | **7.39** | **0.007** |
| Teaching experience | -0.134 | 0.202 | 1 | 0.46 | 0.500 |
| Formal training in training | -0.381 | 0.270 | 1 | 1.90 | 0.168 |
| Student | NA |  |  |  |  |
| Topic | NA |  |  |  |  |
|  |  |  |  |  |  |
| Degree in broadening my perspective on related topics | | | |  |  |
| **Lesson type (SG)** | **0.659** | **0.200** | **2** | **16.81** | **<0.001** |
| **Lesson type (EG)** | **0.436** | **0.140** |  |  |  |
| **Year** | **0.536** | **0.261** | **1** | **4.17** | **0.041** |
| Course | 0.385 | 0.281 | 1 | 1.83 | 0.176 |
| Age | 0.133 | 0.138 | 1 | 0.92 | 0.337 |
| Gender | 0.226 | 0.282 | 1 | 0.64 | 0.425 |
| Teaching experience | 0.178 | 0.277 | 1 | 0.41 | 0.520 |
| Formal training in training | -0.255 | 0.374 | 1 | 0.46 | 0.497 |
| Student | 0.807 |  |  |  |  |
| Topic | 0.237 |  |  |  |  |
|  |  |  |  |  |  |
| Degree of nurturing creative thinking | |  |  |  |  |
| **Lesson type (SG)** | **1.178** | **0.247** | **2** | **19.60** | **<0.001** |
| **Lesson type (EG)** | **1.381** | **0.204** |  |  |  |
| Year | 0.419 | 0.186 | 1 | 4.79 | 0.029 |
| Course | 0.157 | 0.191 | 1 | 0.94 | 0.332 |
| Age | -0.007 | 0.098 | 1 | 0.01 | 0.930 |
| Gender | 0.041 | 0.202 | 1 | 0.07 | 0.788 |
| Teaching experience | 0.298 | 0.199 | 1 | 2.21 | 0.137 |
| **Formal training in training** | **-0.946** | **0.268** | **1** | **12.98** | **<0.001** |
| Student | NA |  |  |  |  |
| Topic | NA |  |  |  |  |
|  |  |  |  |  |  |
| Level of challenge |  |  |  |  |  |
| **Lesson type (SG)** | **0.949** | **0.184** | **2** | **78.79** | **<0.001** |
| **Lesson type (EG)** | **1.153** | **0.149** |  |  |  |
| **Year** | **0.662** | **0.223** | **1** | **8.55** | **0.003** |
| **Course** | **0.738** | **0.241** | **1** | **9.00** | **0.003** |
| Age | 0.112 | 0.116 | 1 | 0.91 | 0.339 |
| Gender | -0.026 | 0.236 | 1 | 0.01 | 0.913 |
| Teaching experience | 0.173 | 0.235 | 1 | 0.55 | 0.460 |
| Formal training in training | -0.060 | 0.315 | 1 | 0.04 | 0.848 |
| Student | 12.000 |  |  |  |  |
| Topic | 18.000 |  |  |  |  |
|  |  |  |  |  |  |
| Class dynamics | | | | | |
| Amount of my attention retained for the length of the tutorial | | | | |  |
| **Lesson type (SG)** | **0.710** | **0.164** | **2** | **41.43** | **<0.001** |
| **Lesson type (EG)** | **0.732** | **0.132** |  |  |  |
| Year | 0.145 | 0.199 | 1 | 0.53 | 0.466 |
| Course | 0.082 | 0.212 | 1 | 0.15 | 0.698 |
| Age | 0.011 | 0.106 | 1 | 0.01 | 0.914 |
| **Gender** | **-0.011** | **0.217** | **1** | **0.00** | **0.960** |
| Teaching experience | 0.342 | 0.216 | 1 | 2.47 | 0.116 |
| **Formal training in training** | **-0.773** | **0.286** | **1** | **6.86** | **0.009** |
| Student | 0.573 |  |  |  |  |
| Topic | 0.094 |  |  |  |  |
|  |  |  |  |  |  |
| Level of engagement with the tutor | |  |  |  |  |
| **Lesson type (SG)** | **0.892** | **0.178** | **2** | **49.55** | **<0.001** |
| **Lesson type (EG)** | **0.817** | **0.132** |  |  |  |
| **Year** | **0.541** | **0.237** | **1** | **5.08** | **0.024** |
| Course | -0.212 | 0.252 | 1 | 0.70 | 0.401 |
| Age | 0.048 | 0.125 | 1 | 0.15 | 0.702 |
| Gender | 0.027 | 0.257 | 1 | 0.01 | 0.915 |
| **Teaching experience** | **0.105** | **0.254** | **1** | **0.17** | **0.680** |
| Formal training in training | -0.543 | 0.339 | 1 | 2.50 | 0.114 |
| Student | 0.720 |  |  |  |  |
| Topic | 0.152 |  |  |  |  |
|  |  |  |  |  |  |
| Level of engagement with other students | |  |  |  |  |
| **Lesson type (SG)** | **1.156** | **0.183** | **2** | **103.67** | **<0.001** |
| **Lesson type (EG)** | **1.257** | **0.138** |  |  |  |
| Year | 0.382 | 0.216 | 1 | 3.07 | 0.080 |
| Course | 0.038 | 0.230 | 1 | 0.03 | 0.868 |
| Age | -0.070 | 0.114 | 1 | 0.38 | 0.539 |
| Gender | 0.221 | 0.235 | 1 | 0.88 | 0.348 |
| Teaching experience | 0.228 | 0.232 | 1 | 0.95 | 0.329 |
| Formal training in training | -0.512 | 0.310 | 1 | 2.67 | 0.102 |
| Student | 0.640 |  |  |  |  |
| Topic | 0.132 |  |  |  |  |
|  |  |  |  |  |  |
| Degree of encouragement to ask questions | | |  |  |  |
| Lesson type (SG) | 0.744 | 0.334 | 2 | 1.42 | 0.492 |
| Lesson type (EG) | 0.417 | 0.241 |  |  |  |
| Year | 0.202 | 0.328 | 1 | 0.37 | 0.542 |
| Course | -0.006 | 0.259 | 1 | 0.00 | 0.971 |
| Age | 0.121 | 0.123 | 1 | 0.98 | 0.321 |
| **Gender** | **0.636** | **0.255** | **1** | **6.15** | **0.013** |
| Teaching experience | 0.211 | 0.248 | 1 | 0.66 | 0.417 |
| **Formal training in training** | **-0.768** | **0.312** | **1** | **6.05** | **0.014** |
| Student | NA |  |  |  |  |
| Topic | NA |  |  |  |  |
|  |  |  |  |  |  |
| Degree of learning from my peers | |  |  |  |  |
| **Lesson type (SG)** | **0.311** | **0.201** | **2** | **20.94** | **<0.001** |
| **Lesson type (EG)** | **0.683** | **0.151** |  |  |  |
| Year | 0.069 | 0.444 | 1 | 0.02 | 0.875 |
| Course | 0.632 | 0.395 | 1 | 2.38 | 0.123 |
| Age | -0.216 | 0.179 | 1 | 1.44 | 0.230 |
| Gender | 0.513 | 0.363 | 1 | 1.92 | 0.166 |
| Teaching experience | 0.425 | 0.354 | 1 | 1.39 | 0.239 |
| Formal training in training | -0.412 | 0.443 | 1 | 0.85 | 0.355 |
| Student | 0.818 |  |  |  |  |
| Student\|Lesson type (SG) | 0.000 |  |  |  |  |
| Student\|Lesson type (EG) | 0.000 |  |  |  |  |
| Topic | 0.000 |  |  |  |  |
|  |  |  |  |  |  |
| Degree of connection with my peers | |  |  |  |  |
| **Lesson type (SG)** | **0.399** | **0.199** | **2** | **31.90** | **<0.001** |
| **Lesson type (EG)** | **0.851** | **0.153** |  |  |  |
| Year | -0.161 | 0.396 | 1 | 0.16 | 0.686 |
| Course | 0.416 | 0.348 | 1 | 1.36 | 0.243 |
| Age | -0.101 | 0.158 | 1 | 0.41 | 0.523 |
| Gender | 0.532 | 0.322 | 1 | 2.60 | 0.107 |
| Teaching experience | 0.338 | 0.314 | 1 | 1.12 | 0.289 |
| Formal training in training | -0.418 | 0.391 | 1 | 1.12 | 0.289 |
| Student | 0.701 |  |  |  |  |
| Topic | 0.000 |  |  |  |  |
|  |  |  |  |  |  |
| Intrinsic motivation parameters | | | | | |
| Interest/enjoyment |  |  |  |  |  |
| **Lesson type (SG)** | **0.469** | **0.218** | **2** | **17.96** | **<0.001** |
| **Lesson type (EG)** | **0.624** | **0.153** |  |  |  |
| Course | -0.046 | 0.142 | 1 | 0.10 | 0.747 |
| Age | 0.240 | 0.290 | 1 | 0.67 | 0.412 |
| Gender | -0.252 | 0.275 | 1 | 0.83 | 0.363 |
| Teaching experience | -0.557 | 0.333 | 1 | 2.67 | 0.102 |
| Formal training in training | -0.106 | 0.277 | 1 | 0.15 | 0.703 |
| Student | 0.516 |  |  |  |  |
| Topic | 0.158 |  |  |  |  |
|  |  |  |  |  |  |
| Perceived competence |  |  |  |  |  |
| Lesson type (SG) | -0.006 | 0.222 | 2 | 1.13 | 0.567 |
| Lesson type (EG) | 0.152 | 0.152 |  |  |  |
| Course | -0.044 | 0.179 | 1 | 0.06 | 0.807 |
| Age | -0.007 | 0.365 | 1 | 0.00 | 0.985 |
| Gender | -0.060 | 0.347 | 1 | 0.03 | 0.864 |
| Teaching experience | -0.524 | 0.420 | 1 | 1.52 | 0.218 |
| Formal training in training | -0.377 | 0.349 | 1 | 1.15 | 0.284 |
| Student | 0.698 |  |  |  |  |
| Topic | 0.193 |  |  |  |  |
|  |  |  |  |  |  |
| Perceived choice |  |  |  |  |  |
| Lesson type (SG) | -0.089 | 0.224 | 2 | 0.37 | 0.830 |
| Lesson type (EG) | 0.048 | 0.152 |  |  |  |
| Course | -0.045 | 0.286 | 1 | 0.02 | 0.875 |
| Age | 0.592 | 0.581 | 1 | 1.01 | 0.314 |
| Gender | 1.066 | 0.557 | 1 | 3.41 | 0.065 |
| Teaching experience | -0.605 | 0.672 | 1 | 0.80 | 0.371 |
| Formal training in training | 0.312 | 0.550 | 1 | 0.32 | 0.573 |
| Student | 1.187 |  |  |  |  |
| Topic | 0.181 |  |  |  |  |
|  |  |  |  |  |  |
| Pressure or tension |  |  |  |  |  |
| Lesson type (SG) | 0.069 | 0.246 | 2 | 0.84 | 0.656 |
| Lesson type (EG) | 0.147 | 0.160 |  |  |  |
| Course | -0.285 | 0.169 | 1 | 2.70 | 0.100 |
| Age | 0.213 | 0.345 | 1 | 0.38 | 0.537 |
| Gender | -0.439 | 0.328 | 1 | 1.73 | 0.189 |
| Teaching experience | -0.244 | 0.396 | 1 | 0.38 | 0.540 |
| **Formal training in training** | **0.740** | **0.337** | **1** | **4.56** | **0.033** |
| Student | 0.649 |  |  |  |  |
| Topic | 0.293 |  |  |  |  |
|  |  |  |  |  |  |
| Bondedness |  |  |  |  |  |
| **Lesson type (SG)** | **1.180** | **0.386** | **2** | **9.55** | **0.008** |
| **Lesson type (EG)** | **0.469** | **0.483** |  |  |  |
| Age | -0.119 | 1.077 | 1 | 0.01 | 0.912 |
| Gender | 0.131 | 0.545 | 1 | 0.06 | 0.810 |
| How well you know | -0.219 | 0.133 | 1 | 2.80 | 0.094 |
| Student | 2.640 |  |  |  |  |
| Rated student | 0.808 |  |  |  |  |
| Topic | 2.272 |  |  |  |  |
| Repeated measure | 0.000 |  |  |  |  |
|  |  |  |  |  |  |
| Overall rating | | | | | |
| Personality |  |  |  |  |  |
| Agreeableness (DI) | 0.008 | 0.011 | 2 | 2.60 | 0.272 |
| Agreeableness (SG) | -0.010 | 0.011 |  |  |  |
| Agreeableness (EG) | 0.008 | 0.009 |  |  |  |
| Conscientiouness (DI) | 0.006 | 0.009 | 2 | 3.88 | 0.144 |
| Conscientiouness (SG) | -0.016 | 0.009 |  |  |  |
| Conscientiouness (EG) | 0.000 | 0.007 |  |  |  |
| Extroversion (DI) | 0.003 | 0.009 | 2 | 2.14 | 0.343 |
| Extroversion (SG) | -0.006 | 0.009 |  |  |  |
| Extroversion (EG) | -0.011 | 0.008 |  |  |  |
| Neuroticism (DI) | -0.002 | 0.009 | 2 | 3.44 | 0.179 |
| Neuroticism (SG) | -0.017 | 0.010 |  |  |  |
| Neuroticism (EG) | 0.000 | 0.008 |  |  |  |
| Openness (DI) | 0.012 | 0.011 | 2 | 0.17 | 0.920 |
| Openness (SG) | 0.005 | 0.012 |  |  |  |
| Openness (EG) | 0.002 | 0.009 |  |  |  |
|  |  |  |  |  |  |
| Learning styles |  |  |  |  |  |
| Reproduction-directed (DI) | 0.017 | 0.006 | 2 | 3.79 | 0.151 |
| Reproduction-directed (SG) | -0.007 | 0.006 |  |  |  |
| Reproduction-directed (EG) | -0.010 | 0.005 |  |  |  |
| Meaning-directed (DI) | 0.009 | 0.004 | 2 | 3.59 | 0.166 |
| Meaning-directed (SG) | -0.002 | 0.004 |  |  |  |
| Meaning-directed (EG) | -0.006 | 0.003 |  |  |  |
| Application-directed (DI) | -0.007 | 0.012 | 2 | 1.51 | 0.469 |
| Application-directed (SG) | 0.010 | 0.012 |  |  |  |
| Application-directed (EG) | -0.005 | 0.010 |  |  |  |
| **Undirected (DI)** | **0.009** | **0.004** | **2** | **7.29** | **0.026** |
| **Undirected (SG)** | **-0.004** | **0.004** |  |  |  |
| **Undirected (EG)** | **-0.009** | **0.004** |  |  |  |
