# Supplementary material for: The effect of using games in teaching conservation
Source: PeerJ. 2018 Apr 30;6:e4509. doi: 10.7717/peerj.4509 (PMC5936071; doi:10.7717/peerj.4509)
Supplement: Supplemental Information 6 — DI–Didactic Instruction; SG–Supplemental Game; EG–Experiential Game. Bold values identify variables that are significant. The second column “Estimate’” shows the estimated differences in mean scores between the two lesson types. Significant variables are highlighted in bold. [file peerj-06-4509-s006.docx]

Supplementary Table S2. Table of post-hoc results of perception analysed using ordinal logistic models. DI – Didactic Instruction; SG – Supplemental Game; EG – Experiential Game. Bold values identify variables that are significant. The second column “Estimate’” shows the estimated differences in mean scores between the two lesson types. Significant variables are highlighted in bold.

|  | Estimate | SE | Z | P |
| --- | --- | --- | --- | --- |
| Knowledge acquisition | | | | |
| Amount of content taught | |  |  |  |
| DI - SG | -1.43 | 0.208 | 0.98 | 0.590 |
| DI - EG | -1.61 | 0.244 | 1.97 | 0.121 |
| SG - EG | -1.69 | 0.213 | 0.45 | 0.894 |
|  |  |  |  |  |
| Degree of remembrance of content | | |  |  |
| **DI - SG** | **-1.07** | **0.157** | **3.24** | **0.003** |
| **DI - EG** | **-1.65** | **0.200** | **3.86** | **<0.001** |
| SG - EG | -1.58 | 0.165 | -0.38 | 0.922 |
|  |  |  |  |  |
| Level of understanding of the topic | | |  |  |
| **DI - SG** | **-1.23** | **0.177** | **2.49** | **0.034** |
| DI - EG | -1.70 | 0.224 | 1.96 | 0.122 |
| SG - EG | -1.49 | 0.182 | -1.07 | 0.534 |
|  |  |  |  |  |
| Degree of appreciation of application of topic | | |  |  |
| DI - SG | -0.96 | 0.243 | 0.58 | 0.830 |
| **DI - EG** | **-1.09** | **0.288** | **2.62** | **0.024** |
| SG - EG | -1.40 | 0.251 | 1.31 | 0.388 |
|  |  |  |  |  |
| Development | | | | |
| Level of motivation for me to learn more after the lesson | | | |  |
| **DI - SG** | **-1.63** | **0.188** | **3.39** | **0.002** |
| **DI - EG** | **-2.16** | **0.220** | **5.08** | **<0.001** |
| SG - EG | -2.26 | 0.202 | 0.67 | 0.778 |
|  |  |  |  |  |
| Degree in broadening my perspective on related topics | | | |  |
| **DI - SG** | **-0.68** | **0.212** | **3.30** | **<0.001** |
| **DI - EG** | **-1.34** | **0.254** | **3.11** | **<0.001** |
| SG - EG | -1.11 | 0.216 | -1.14 | 0.492 |
|  |  |  |  |  |
| Degree of nurturing creative thinking | | |  |  |
| **DI - SG** | **-0.71** | **0.150** | **5.71** | **<0.001** |
| **DI - EG** | **-1.58** | **0.186** | **7.80** | **<0.001** |
| SG - EG | -1.66 | 0.163 | 0.55 | 0.844 |
|  |  |  |  |  |
| Level of challenge |  |  |  |  |
| **DI - SG** | **-0.51** | **0.185** | **5.17** | **<0.001** |
| **DI - EG** | **-1.46** | **0.225** | **7.75** | **<0.001** |
| SG - EG | -1.67 | 0.200 | 1.08 | 0.528 |
|  |  |  |  |  |
| Class dynamics | | | | |
| Amount of my attention retained for the length of the tutorial | | | |  |
| **DI - SG** | **-1.04** | **0.168** | **4.33** | **<0.001** |
| **DI - EG** | **-1.75** | **0.203** | **5.54** | **<0.001** |
| SG - EG | -1.77 | 0.179 | 0.14 | 0.989 |
|  |  |  |  |  |
| Level of engagement with the tutor | | |  |  |
| **DI - SG** | **-0.45** | **0.189** | **5.00** | **<0.001** |
| **DI - EG** | **-1.34** | **0.228** | **6.19** | **<0.001** |
| SG - EG | -1.26 | 0.196 | -0.42 | 0.907 |
|  |  |  |  |  |
| Level of engagement with other students | | |  |  |
| **DI - SG** | **-0.83** | **0.177** | **6.33** | **<0.001** |
| **DI - EG** | **-1.99** | **0.228** | **9.08** | **<0.001** |
| SG - EG | -2.09 | 0.197 | 0.58 | 0.829 |
|  |  |  |  |  |
| Degree of encouragement to ask questions | | |  |  |
| **DI - SG** | **-1.30** | **0.239** | **2.79** | **0.014** |
| **DI - EG** | **-1.88** | **0.286** | **2.35** | **0.049** |
| SG - EG | -1.65 | 0.239 | -1.14 | 0.488 |
|  |  |  |  |  |
| Degree of learning from my peers | | |  |  |
| DI - SG | -1.30 | 0.272 | 1.55 | 0.269 |
| **DI - EG** | **-1.61** | **0.308** | **4.53** | **<0.001** |
| SG - EG | -1.98 | 0.279 | 1.84 | 0.156 |
|  |  |  |  |  |
| Degree of connection with my peers | | |  |  |
| DI - SG | -1.30 | 0.247 | 2.00 | 0.112 |
| **DI - EG** | **-1.70** | **0.287** | **5.56** | **<0.001** |
| **SG - EG** | **-2.15** | **0.258** | **2.25** | **0.063** |
|  |  |  |  |  |
| Intrinsic motivation parameters | | | | |
| Interest/enjoyment |  |  |  |  |
| DI - SG | -0.10 | 0.191 | 2.15 | 0.080 |
| **DI - EG** | **-0.57** | **0.246** | **4.08** | **<0.001** |
| SG - EG | -0.73 | 0.195 | 0.70 | 0.763 |
|  |  |  |  |  |
| Perceived competence | |  |  |  |
| DI - SG | 0.17 | 0.234 | -0.03 | 1.000 |
| DI - EG | 0.17 | 0.281 | 1.00 | 0.578 |
| SG - EG | 0.01 | 0.234 | 0.70 | 0.766 |
|  |  |  |  |  |
| Perceived choice |  |  |  |  |
| DI - SG | -0.37 | 0.355 | -0.40 | 0.916 |
| DI - EG | -0.28 | 0.389 | 0.32 | 0.946 |
| SG - EG | -0.42 | 0.355 | 0.60 | 0.823 |
|  |  |  |  |  |
| Pressure or tension |  |  |  |  |
| DI - SG | 0.77 | 0.234 | 0.28 | 0.958 |
| DI - EG | 0.70 | 0.292 | 0.92 | 0.630 |
| SG - EG | 0.62 | 0.233 | 0.31 | 0.949 |
|  |  |  |  |  |
| Bondedness |  |  |  |  |
| DI - SG | 0.20 | 0.591 | 0.97 | 0.595 |
| **DI - EG** | **1.38** | **0.600** | **3.05** | **0.006** |
| SG - EG | 0.91 | 0.675 | -1.44 | 0.319 |
