# Supplementary material for: The effect of using games in teaching conservation
Source: PeerJ. 2018 Apr 30;6:e4509. doi: 10.7717/peerj.4509 (PMC5936071; doi:10.7717/peerj.4509)
Supplement: Supplemental Information 7 — The probability of behaviour was analysed with a Generalised linear mixed model with Binomial error distribution and frequency of occurrence was analysed with a Generalised linear mixed model with Poisson error distribution. DI–Didactic Instruction; SG–Supplemental Game; EG–Experiential Game; E.score–Extroversion score. Definition of other explanatory variables [reference level]: Age–age of student as an ordinal variable; Gender–gender of student [Female]; Duration–duration of lesson; Year–year of course [2015]; Course–Diploma in Oxford or Wildlife Conservation Course in Malaysia [Diploma]; Topic–topic of lesson. Student and topic were entered as random variables. The second column shows either (i) the estimate of the slope for a continuous fixed variable, (ii) the estimate of the difference in mean from the reference level for a discrete fixed variable, or (iii) the variance of a random variable. Significant variables are highlighted in bold. [file peerj-06-4509-s007.docx]

Supplementary Table S3. Table of behavioural results. The probability of behaviour was analysed with a Generalised linear mixed model with Binomial error distribution and frequency of occurrence was analysed with a Generalised linear mixed model with Poisson error distribution. DI – Didactic Instruction; SG – Supplemental Game; EG – Experiential Game; E.score – Extroversion score. Definition of other explanatory variables [reference level]: Age – age of student as an ordinal variable; Gender – gender of student [Female]; Duration – duration of lesson; Year – year of course [2015]; Course – Diploma in Oxford or Wildlife Conservation Course in Malaysia [Diploma]; Topic – topic of lesson. Student and topic were entered as random variables. The second column shows either (i) the estimate of the slope for a continuous fixed variable, (ii) the estimate of the difference in mean from the reference level for a discrete fixed variable, or (iii) the variance of a random variable. Significant variables are highlighted in bold.

|  | Estimate (fixed)/ Variance (random) | SE | Df | Chisq | Pr(>Chi) |
| --- | --- | --- | --- | --- | --- |
| Asking questions | | | | | |
| Probability of occurrence |  |  |  |  |  |
| E.score*Lesson type (SG) | -0.028 | 0.047 | 2 | 0.36 | 0.837 |
| E.score*Lesson type (EG) | -0.008 | 0.039 |  |  |  |
| E.score | 0.006 | 0.036 | 1 | 0.01 | 0.915 |
| **Lesson type (SG)** | **0.134** | **1.071** | **2** | **6.08** | **0.048** |
| **Lesson type (EG)** | **-0.818** | **0.903** |  |  |  |
| Age | 0.023 | 0.178 | 1 | 0.02 | 0.894 |
| Gender | -0.097 | 0.385 | 1 | 0.06 | 0.804 |
| **Duration** | **0.018** | **0.005** | **1** | **15.29** | **<0.001** |
| Year | 0.097 | 0.377 | 1 | 0.06 | 0.802 |
| Course | 0.570 | 0.432 | 1 | 1.80 | 0.180 |
| Student | 1.072 |  |  |  |  |
| Topic | 0.121 |  |  |  |  |
|  |  |  |  |  |  |
| Frequency of occurrence |  |  |  |  |  |
| E.score*Lesson type (SG) | 0.000 | 0.019 | 2 | 0.27 | 0.873 |
| E.score*Lesson type (EG) | -0.006 | 0.015 |  |  |  |
| E.score | 0.006 | 0.011 | 1 | 0.24 | 0.626 |
| Lesson type (SG) | 0.108 | 0.431 | 2 | 1.31 | 0.519 |
| Lesson type (EG) | 0.350 | 0.377 |  |  |  |
| Age | 0.043 | 0.052 | 1 | 0.80 | 0.372 |
| Gender | -0.146 | 0.116 | 1 | 1.73 | 0.188 |
| **Duration** | **0.081** | **0.145** | **1** | **16.97** | **<0.001** |
| **Year** | **-0.370** | **0.141** | **1** | **7.65** | **0.006** |
| Course | 0.081 | 0.145 | 1 | 0.49 | 0.485 |
| Student | 0.044 |  |  |  |  |
| Topic | 0.057 |  |  |  |  |
|  |  |  |  |  |  |
| Answering questions | | | | | |
| Probability of occurrence |  |  |  |  |  |
| E.score*Lesson type (SG) | -0.029 | 0.048 | 2 | 0.68 | 0.712 |
| E.score*Lesson type (EG) | -0.031 | 0.040 |  |  |  |
| E.score | 0.004 | 0.037 | 1 | 0.19 | 0.659 |
| Lesson type (SG) | 0.039 | 1.128 | 2 | 1.34 | 0.513 |
| Lesson type (EG) | 0.301 | 0.990 |  |  |  |
| Age | 0.000 | 0.184 | 1 | 0.00 | 0.995 |
| Gender | -0.712 | 0.403 | 1 | 3.04 | 0.081 |
| Duration | -0.001 | 0.006 | 1 | 0.04 | 0.838 |
| Year | 1.049 | 0.453 | 1 | 5.99 | 0.014 |
| **Course** | **-1.063** | **0.507** | **1** | **4.86** | **0.028** |
| Student | 1.197 |  |  |  |  |
| Topic | 1.118 |  |  |  |  |
|  |  |  |  |  |  |
| Joyful behaviour | | | | | |
| Probability of occurrence |  |  |  |  |  |
| **E.score*Lesson type (SG)** | **0.029** | **0.123** | **2** | **2.20** | **0.334** |
| **E.score*Lesson type (EG)** | **-0.099** | **0.074** |  |  |  |
| E.score | 0.003 | 0.047 | 1 | 0.77 | 0.381 |
| **Lesson type (SG)** | **8.002** | **3.121** | **2** | **43.42** | **<0.001** |
| **Lesson type (EG)** | **8.480** | **2.664** |  |  |  |
| Age | 0.225 | 0.227 | 1 | 1.10 | 0.295 |
| **Gender** | **-1.138** | **0.449** | **1** | **6.62** | **0.010** |
| **Duration** | **-0.078** | **0.025** | **1** | **14.72** | **<0.001** |
| **Year** | **4.096** | **1.060** | **1** | **30.71** | **<0.001** |
| **Course** | **1.443** | **0.786** | **1** | **3.28** | **0.070** |
| Student | 0.094 |  |  |  |  |
| Topic | 24.649 |  |  |  |  |
|  |  |  |  |  |  |
| Frequency of occurrence |  |  |  |  |  |
| E.score*Lesson type (SG) | -0.003 | 0.013 | 2 | 3.12 | 0.210 |
| E.score*Lesson type (EG) | -0.018 | 0.011 |  |  |  |
| E.score | -0.003 | 0.014 | 1 | 0.76 | 0.382 |
| **Lesson type (SG)** | **0.225** | **0.318** | **2** | **76.93** | **<0.001** |
| **Lesson type (EG)** | **1.640** | **0.270** |  |  |  |
| Age | 0.032 | 0.077 | 1 | 0.19 | 0.660 |
| **Gender** | **-0.340** | **0.165** | **1** | **4.51** | **0.034** |
| **Duration** | **-0.013** | **0.002** | **1** | **58.20** | **<0.001** |
| Year | 0.340 | 0.156 | 1 | 4.63 | 0.031 |
| **Course** | **0.731** | **0.190** | **1** | **13.41** | **<0.001** |
| Student | 0.277 |  |  |  |  |
| Topic | 0.251 |  |  |  |  |
|  |  |  |  |  |  |
| Distraction | | | | | |
| Probability of occurrence |  |  |  |  |  |
| E.score*Lesson type (SG) | 0.034 | 0.042 | 2 | 0.66 | 0.717 |
| E.score*Lesson type (EG) | 0.000 | 0.000 |  |  |  |
| E.score | -0.009 | 0.037 | 1 | 0.03 | 0.857 |
| **Lesson type (SG)** | **-1.206** | **1.166** | **2** | **7.12** | **0.028** |
| **Lesson type (EG)** | **-1.615** | **0.958** |  |  |  |
| Age | 0.095 | 0.181 | 1 | 0.28 | 0.597 |
| Gender | -0.075 | 0.393 | 1 | 0.04 | 0.844 |
| Duration | 0.015 | 0.053 | 1 | 0.11 | 0.741 |
| Course | 0.002 | 0.004 | 1 | 1.66 | 0.198 |
| Student | 1.083 |  |  |  |  |
| Topic | 0.050 |  |  |  |  |
|  |  |  |  |  |  |
| Frequency of occurrence |  |  |  |  |  |
| E.score*Lesson type (SG) | -0.010 | 0.016 | 2 | 2.36 | 0.308 |
| E.score*Lesson type (EG) | 0.014 | 0.013 |  |  |  |
| E.score | 0.002 | 0.009 | 1 | 0.76 | 0.384 |
| **Lesson type (SG)** | **0.034** | **0.362** | **2** | **7.96** | **0.019** |
| **Lesson type (EG)** | **-0.602** | **0.309** |  |  |  |
| Age | 0.021 | 0.038 | 1 | 0.32 | 0.573 |
| Gender | -0.037 | 0.087 | 1 | 0.37 | 0.545 |
| **Duration** | **-0.008** | **0.002** | **1** | **21.52** | **<0.001** |
| Year | 0.133 | 0.100 | 1 | 1.80 | 0.179 |
| Course | -0.018 | 0.096 | 1 | 0.04 | 0.845 |
| Student | 0.007 |  |  |  |  |
| Topic | 0.000 |  |  |  |  |
