# Supplementary material for: The effect of using games in teaching conservation
Source: PeerJ. 2018 Apr 30;6:e4509. doi: 10.7717/peerj.4509 (PMC5936071; doi:10.7717/peerj.4509)
Supplement: Supplemental Information 8 — The probability of behaviour was analysed with a Generalised linear mixed model with Binomial error distribution and frequency of occurrence was analysed with a Generalised linear mixed model with Poisson error distribution. DI–Didactic Instruction; SG–Supplemental Game; EG–Experiential Game. The second column “Estimate’” shows the estimated differences in mean scores between the two lesson types. Significant variables are highlighted in bold. [file peerj-06-4509-s008.docx]

Supplementary Table S4. Table of post-hoc behavioural results. The probability of behaviour was analysed with a Generalised linear mixed model with Binomial error distribution and frequency of occurrence was analysed with a Generalised linear mixed model with Poisson error distribution. DI – Didactic Instruction; SG – Supplemental Game; EG – Experiential Game. The second column “Estimate’” shows the estimated differences in mean scores between the two lesson types. Significant variables are highlighted in bold.

|  | Estimate | SE | Z | P |
| --- | --- | --- | --- | --- |
| Asking questions | | | | |
| Probability of occurrence | | | | |
| DI - SG | -0.229 | 0.294 | 1.22 | 0.442 |
| **DI - EG** | **-0.694** | **0.339** | **2.62** | **0.024** |
| SG - EG | -1.203 | 0.299 | 1.39 | 0.348 |
| Frequency of occurrence | | | | |
| DI - SG | -0.025 | 0.128 | -0.59 | 0.826 |
| DI - EG | 0.079 | 0.143 | -1.15 | 0.485 |
| SG - EG | 0.187 | 0.127 | -0.62 | 0.808 |
|  |  |  |  |  |
| Answering questions | | | | |
| Probability of occurrence | | | | |
| DI - SG | -0.163 | 0.418 | 1.13 | 0.493 |
| DI - EG | -0.741 | 0.504 | 0.64 | 0.797 |
| SG - EG | -0.501 | 0.403 | -0.42 | 0.909 |
|  |  |  |  |  |
| Joyful behaviour | | | | |
| Probability of occurrence | | | | |
| **DI - SG** | **-0.549** | **1.380** | **-4.31** | **<0.001** |
| **DI - EG** | **7.859** | **1.960** | **-3.24** | **0.003** |
| SG - EG | 5.643 | 1.766 | 1.23 | 0.436 |
| Frequency of occurrence | | | | |
| DI - SG | 0.393 | 0.163 | -0.98 | 0.593 |
| **DI - EG** | **0.555** | **0.182** | **-8.59** | **<0.001** |
| **SG - EG** | **1.664** | **0.155** | **-6.49** | **<0.001** |
|  |  |  |  |  |
| Distraction | | | | |
| Probability of occurrence | | | | |
| **DI - SG** | **-0.710** | **0.282** | **2.43** | **0.040** |
| **DI - EG** | **-1.593** | **0.342** | **2.44** | **0.039** |
| SG - EG | -1.599 | 0.300 | 0.02 | 1.000 |
| Frequency of occurrence | | | | |
| DI - SG | 0.236 | 0.074 | 1.47 | 0.323 |
| **DI - EG** | **0.055** | **0.100** | **2.64** | **0.035** |
| SG - EG | -0.068 | 0.083 | 1.02 | 0.572 |
