# Supplementary material for: The effect of using games in teaching conservation
Source: PeerJ. 2018 Apr 30;6:e4509. doi: 10.7717/peerj.4509 (PMC5936071; doi:10.7717/peerj.4509)
Supplement: Supplemental Information 9 — Mean correctness reflect the difficulty level, with values closer to 0 meaning that questions were easier. SD reflects level of discrimination. For example, in the case of pre-lesson, the minimum mean correctness is 0.152 and minimum SD correctness is 0.123, this indicates that 66% of students had scores between +/− 1 SD from the mean (i.e. 0.029–0.275). [file peerj-06-4509-s009.docx]

Supplementary Table S5. Table of mean correctness and standard deviation (SD) of corrected. Mean correctness reflect the difficulty level, with values closer to 0 meaning that questions were easier. SD reflects level of discrimination. For example, in the case of pre-lesson, the minimum mean correctness is 0.152 and minimum SD correctness is 0.123, this indicates that 66% of students had scores between +/- 1 SD from the mean

(i.e. 0.029-0.275)

|  | Before lesson | | | | After lesson | | | | | One-week post lesson | | | | |
| --- | --- | --- | --- | --- | --- | --- | --- | --- | --- | --- | --- | --- | --- | --- |
|  | Min | Max | Mean across lessons | SD across lessons | | Min | Max | Mean across lessons | SD across lessons | | Min | Max | Mean across lessons | SD across lessons |
| Mean correctness of each lesson (difficulty) | 0.152 | 0.578 | 0.351 | 0.102 | | 0.293 | 0.750 | 0.551 | 0.116 | | 0.400 | 0.722 | 0.522 | 0.106 |
| SD correctness of each lesson (discrimination) | 0.123 | 0.283 | 0.209 | 0.052 | | 0.144 | 0.319 | 0.234 | 0.043 | | 0.165 | 0.333 | 0.244 | 0.042 |
