# Supplementary material for: The effect of using games in teaching conservation
Source: PeerJ. 2018 Apr 30;6:e4509. doi: 10.7717/peerj.4509 (PMC5936071; doi:10.7717/peerj.4509)
Supplement: Supplemental Information 10 — Table of learning results as analysed using Generalised Linear Mixed Models with Binomial error distribution. DI–Didactic Instruction; SG–Supplemental Game; EG–Experiential Game. Definition of other explanatory variables [reference level for discrete variables]: BeforeAfter–Before or After lesson; Year–year of course [2015]; Course–Diploma in Oxford or Wildlife Conservation Course in Malaysia [Diploma]; Topic–topic of lesson. The second column shows either (i) the estimate of the slope for a continuous fixed variable, (ii) the estimate of the difference in mean from the reference level for a discrete fixed variable, or (iii) the variance of a random variable. Significant variables are highlighted in bold. [file peerj-06-4509-s010.docx]

Supplementary Table S6. Table of learning results as analysed using Generalised Linear Mixed Models with Binomial error distribution. DI – Didactic Instruction; SG – Supplemental Game; EG – Experiential Game. Definition of other explanatory variables [reference level for discrete variables]: BeforeAfter – Before or After lesson; Year – year of course [2015]; Course – Diploma in Oxford or Wildlife Conservation Course in Malaysia [Diploma]; Topic – topic of lesson. The second column shows either (i) the estimate of the slope for a continuous fixed variable, (ii) the estimate of the difference in mean from the reference level for a discrete fixed variable, or (iii) the variance of a random variable. Significant variables are highlighted in bold.

|  | Estimate (fixed)/ Variance (random) | SE | df | χ^2^ | *p* | |
| --- | --- | --- | --- | --- | --- | --- |
| *All question types* | | | | | |  |
| Both years | | | | | |  |
| (Intercept) | 0.440 | 0.128 |  |  |  | |
| BeforeAfter*Lesson type (SG) | 0.067 | 0.106 | 2 | 5.18 | 0.075 | |
| BeforeAfter*Lesson type (EG) | 0.260 | 0.118 |  |  |  |  |
| **BeforeAfter** | **-1.055** | **0.08** | **1** | **467.08** | **<0.001** | |
| Lesson type (SG) | -0.029 | 0.172 | 2 | 0.43 | 0.808 | |
| Lesson type (EG) | -0.001 | 0.097 |  |  |  |  |
| **Year** | **-0.183** | **0.08** | **1** | **5.01** | **0.025** | |
| Course | -0.177 | 0.093 | 1 | 3.42 | 0.065 | |
| Student | 0 |  |  |  |  | |
| Student\|Lesson type (SG) | 0.318 |  |  |  |  | |
| Student\|Lesson type (EG) | 0.447 |  |  |  |  | |
| Topic | 0.357 |  |  |  |  | |
|  |  |  |  |  |  | |
| Year 2015 | | | | | |  |
| (Intercept) | 0.761 | 0.183 |  |  |  | |
| BeforeAfter*Lesson type (SG) | 0.34 | 0.161 | 2 | 4.71 | 0.095 | |
| BeforeAfter*Lesson type (EG) | 0.141 | 0.158 |  |  |  |  |
| **BeforeAfter** | **-1.209** | **0.124** | **1** | **291.72** | **<0.001** | |
| Lesson type (SG) | -0.148 | 0.22 | 2 | 2.16 | 0.34 | |
| Lesson type (EG) | -0.242 | 0.151 |  |  |  |  |
| **Course** | **-0.419** | **0.175** | **1** | **5.21** | **0.022** | |
| Student | 0 |  |  |  |  | |
| Student\|Lesson type (SG) | 0.392 |  |  |  |  | |
| Student\|Lesson type (EG) | 0.418 |  |  |  |  | |
| Topic | 0.29 |  |  |  |  | |
|  |  |  |  |  |  | |
| Year 2016 | | | | | |  |
| (Intercept) | 0.498 | 0.157 |  |  |  | |
| BeforeAfterLT (Before)*Lesson type (EG) | 0.029 | 0.146 | 4 | 3.77 | 0.438 | |
| BeforeAfterLT (Before)*Lesson type (SG) | 0.282 | 0.2 |  |  |  |  |
| BeforeAfterLT (LT)*Lesson type (EG) | 0.127 | 0.142 |  |  |  |  |
| BeforeAfterLT (LT)*Lesson type (SG) | 0.331 | 0.193 |  |  |  |  |
| **BeforeAfterLT (Before)** | **-0.929** | **0.106** | **2** | **213.96** | **<0.001** | |
| **BeforeAfterLT (LT)** | **-0.177** | **0.103** |  |  |  |  |
| Lesson type (SG) | -0.453 | 0.222 | 2 | 2.77 | 0.25 | |
| Lesson type (EG) | 0.032 | 0.117 |  |  |  |  |
| **Course** | **-0.415** | **0.146** | **1** | **6.38** | **0.012** | |
| Student | 0.339 |  |  |  |  | |
| Student\|Lesson type (SG) | 0.173 |  |  |  |  | |
| Student\|Lesson type (EG) | 0.301 |  |  |  |  | |
| Topic | 0.277 |  |  |  |  | |
|  |  |  |  |  |  | |
| *Reproduction questions* | | | | | |  |
| Both years | | | | | |  |
| (Intercept) | 1.443 | 0.342 |  |  |  | |
| **BeforeAfter*Lesson type (SG)** | **0.387** | **0.166** | **2** | **30.02** | **<0.001** | |
| **BeforeAfter*Lesson type (EG)** | **0.94** | **0.173** |  |  |  |  |
| **BeforeAfter** | **-1.602** | **0.124** | **1** | **307.70** | **<0.001** | |
| Lesson type (SG) | -0.757 | 0.215 | 2 | 2.18 | 0.337 | |
| Lesson type (EG) | -0.219 | 0.138 |  |  |  |  |
| Year | -0.096 | 0.106 | 1 | 0.52 | 0.47 | |
| **Course** | **-0.265** | **0.12** | **1** | **4.66** | **0.031** | |
| Reproduction-directed learning score | -0.01 | 0.005 | 1 | 3.57 | 0.059 | |
| Student | 0 |  |  |  |  | |
| Student\|Lesson type (SG) | 0.345 |  |  |  |  | |
| Student\|Lesson type (EG) | 0.376 |  |  |  |  | |
| Topic | 0.38 |  |  |  |  | |
|  |  |  |  |  |  | |
| Year 2015 | | | | | |  |
| (Intercept) | 2.568 | 0.502 |  |  |  | |
| **BeforeAfter*Lesson type (SG)** | **1.34** | **0.241** | **2** | **33.71** | **<0.001** | |
| **BeforeAfter*Lesson type (EG)** | **0.529** | **0.25** |  |  |  |  |
| **BeforeAfter** | **-1.967** | **0.192** | **1** | **190.43** | **<0.001** | |
| Lesson type (SG) | -0.996 | 0.323 | 2 | 3.52 | 0.172 | |
| Lesson type (EG) | -0.573 | 0.219 |  |  |  |  |
| Course | -0.383 | 0.207 | 1 | 3.29 | 0.07 | |
| **Reproduction-directed learning score** | **-0.023** | **0.008** | **1** | **9.20** | **0.002** | |
| Student | 0 |  |  |  |  | |
| Student\|Lesson type (SG) | 0.327 |  |  |  |  | |
| Student\|Lesson type (EG) | 0.387 |  |  |  |  | |
| Topic | 0.43 |  |  |  |  | |
|  |  |  |  |  |  | |
| Year 2016 | | | | | |  |
| (Intercept) | 0.331 | 0.417 |  |  |  | |
| BeforeAfterLT (Before)*Lesson type (EG) | 0.323 | 0.225 | 4 | 4.40 | 0.355 | |
| BeforeAfterLT (Before)*Lesson type (SG) | 0.55 | 0.277 |  |  |  |  |
| BeforeAfterLT (LT)*Lesson type (EG) | 0.175 | 0.219 |  |  |  |  |
| BeforeAfterLT (LT)*Lesson type (SG) | 0.303 | 0.265 |  |  |  |  |
| **BeforeAfterLT (Before)** | **-1.322** | **0.165** | **2** | **150.81** | **<0.001** | |
| **BeforeAfterLT (LT)** | **-0.191** | **0.157** |  |  |  |  |
| Lesson type (SG) | -0.646 | 0.297 | 2 | 3.63 | 0.163 | |
| Lesson type (EG) | -0.045 | 0.178 |  |  |  |  |
| Course | -0.257 | 0.132 | 1 | 3.53 | 0.06 | |
| Reproduction-directed learning score | -0.056 | 0.097 | 1 | 0.51 | 0.475 | |
| Student | 0 |  |  |  |  | |
| Student\|Lesson type (SG) | 0.307 |  |  |  |  | |
| Student\|Lesson type (EG) | 0.446 |  |  |  |  | |
| Topic | 0.315 |  |  |  |  | |
|  |  |  |  |  |  | |
| *Meaning questions* | | | | | |  |
| Both years | | | | | |  |
| (Intercept) | 0.139 | 0.484 |  |  |  | |
| BeforeAfter*Lesson type (SG) | -0.207 | 0.166 | 2 | 5.34 | 0.069 | |
| BeforeAfter*Lesson type (EG) | -0.457 | 0.2 |  |  |  |  |
| **BeforeAfter** | **-0.667** | **0.128** | **1** | **147.08** | **<0.001** | |
| Lesson type (SG) | 0.484 | 0.26 | 2 | 3.13 | 0.209 | |
| Lesson type (EG) | -0.047 | 0.146 |  |  |  |  |
| **Year** | **-0.284** | **0.111** | **1** | **6.68** | **0.01** | |
| Course | -0.25 | 0.133 | 1 | 3.47 | 0.063 | |
| Meaning-directed learning score | 0.001 | 0.003 | 1 | 0.11 | 0.737 | |
| Student | 0 |  |  |  |  | |
| Student\|Lesson type (SG) | 0.225 |  |  |  |  | |
| Student\|Lesson type (EG) | 0.287 |  |  |  |  | |
| Topic | 0.57 |  |  |  |  | |
|  |  |  |  |  |  | |
| Year 2015 | | | | | |  |
| (Intercept) | -0.324 | 0.822 |  |  |  | |
| BeforeAfter*Lesson type (SG) | -0.503 | 0.274 | 2 | 3.99 | 0.136 | |
| BeforeAfter*Lesson type (EG) | -0.161 | 0.261 |  |  |  |  |
| **BeforeAfter** | **-0.696** | **0.214** | **1** | **92.25** | **<0.001** | |
| Lesson type (SG) | 0.391 | 0.354 | 2 | 5.87 | 0.053 | |
| Lesson type (EG) | -0.348 | 0.244 |  |  |  |  |
| **Course** | **-0.52** | **0.228** | **1** | **4.90** | **0.027** | |
| Meaning-directed learning score | 0.005 | 0.005 | 1 | 1.11 | 0.293 | |
| Student | 0 |  |  |  |  | |
| Student\|Lesson type (SG) | 0.288 |  |  |  |  | |
| Student\|Lesson type (EG) | 0.247 |  |  |  |  | |
| Topic | 0.491 |  |  |  |  | |
|  |  |  |  |  |  | |
| Year 2016 | | | | | |  |
| (Intercept) | 1.214 | 0.613 |  |  |  | |
| BeforeAfterLT (Before)*Lesson type (EG) | -0.25 | 0.223 | 4 | 7.06 | 0.133 | |
| BeforeAfterLT (Before)*Lesson type (SG) | -0.439 | 0.43 |  |  |  |  |
| BeforeAfterLT (LT)*Lesson type (EG) | 0.132 | 0.216 |  |  |  |  |
| BeforeAfterLT (LT)*Lesson type (SG) | 0.554 | 0.418 |  |  |  |  |
| **BeforeAfterLT (Before)** | **-0.656** | **0.162** | **2** | **65.75** | **<0.001** | |
| **BeforeAfterLT (LT)** | **-0.256** | **0.159** |  |  |  |  |
| Lesson type (SG) | 0.14 | 0.439 | 2 | 0.37 | 0.83 | |
| Lesson type (EG) | -0.01 | 0.178 |  |  |  |  |
| Course | -0.277 | 0.188 | 1 | 2.33 | 0.127 | |
| Meaning-directed learning score | -0.15 | 0.104 | 1 | 2.62 | 0.106 | |
| Student | 0.322 |  |  |  |  | |
| Student\|Lesson type (SG) | 0.177 |  |  |  |  | |
| Student\|Lesson type (EG) | 0.645 |  |  |  |  | |
| Topic | 0.479 |  |  |  |  | |
|  |  |  |  |  |  | |
| *Application questions* | | | | | |  |
| Both years | | | | | |  |
| (Intercept) | -0.222 | 0.887 |  |  |  | |
| BeforeAfter*Lesson type (SG) | -0.222 | 0.288 | 2 | 0.91 | 0.635 | |
| BeforeAfter*Lesson type (EG) | 0.029 | 0.307 |  |  |  |  |
| **BeforeAfter** | **-0.638** | **0.213** | **1** | **35.91** | **<0.001** | |
| Lesson type (SG) | -0.124 | 0.418 | 2 | 5.25 | 0.072 | |
| Lesson type (EG) | 0.539 | 0.247 |  |  |  |  |
| Year | -0.44 | 0.229 | 1 | 3.60 | 0.058 | |
| **Course** | **-0.596** | **0.262** | **1** | **5.15** | **0.023** | |
| Application-directed learning score | 0.016 | 0.016 | 1 | 0.96 | 0.327 | |
| Student | 0.494 |  |  |  |  | |
| Student\|Lesson type (SG) | 0.456 |  |  |  |  | |
| Student\|Lesson type (EG) | 0.9 |  |  |  |  | |
| Topic | 0.725 |  |  |  |  | |
|  |  |  |  |  |  | |
| Year 2015 | | | | | |  |
| (Intercept) | -0.201 | 1.186 |  |  |  | |
| BeforeAfter*Lesson type (SG) | -0.482 | 0.416 | 2 | 1.60 | 0.45 | |
| BeforeAfter*Lesson type (EG) | -0.41 | 0.387 |  |  |  |  |
| **BeforeAfter** | **-0.467** | **0.299** | **1** | **25.14** | **<0.001** | |
| Lesson type (SG) | 0.524 | 0.473 | 2 | 1.68 | 0.431 | |
| Lesson type (EG) | 0.517 | 0.323 |  |  |  |  |
| **Course** | **-0.87** | **0.379** | **1** | **5.04** | **0.025** | |
| Application-directed learning score | 0.012 | 0.022 | 1 | 0.33 | 0.567 | |
| Student | 0.442 |  |  |  |  | |
| Student\|Lesson type (SG) | 0.239 |  |  |  |  | |
| Student\|Lesson type (EG) | 0.791 |  |  |  |  | |
| Topic | 0.515 |  |  |  |  | |
|  |  |  |  |  |  | |
| Year 2016 | | | | | |  |
| (Intercept) | -1.298 | 1.103 |  |  |  | |
| BeforeAfterLT (Before)*Lesson type (EG) | -0.155 | 0.432 | 4 | 4.90 | 0.298 | |
| BeforeAfterLT (Before)*Lesson type (SG) | 0.733 | 0.49 |  |  |  |  |
| BeforeAfterLT (LT)*Lesson type (EG) | -0.014 | 0.427 |  |  |  |  |
| BeforeAfterLT (LT)*Lesson type (SG) | -0.11 | 0.495 |  |  |  |  |
| **BeforeAfterLT (Before)** | **-0.655** | **0.299** | **2** | **14.52** | **<0.001** | |
| **BeforeAfterLT (LT)** | **0.161** | **0.293** |  |  |  |  |
| Lesson type (SG) | -1.135 | 0.717 | 2 | 4.35 | 0.114 | |
| Lesson type (EG) | 0.383 | 0.375 |  |  |  |  |
| Course | -0.292 | 0.287 | 1 | 1.02 | 0.312 | |
| Application-directed learning score | 0.134 | 0.186 | 1 | 1.95 | 0.163 | |
| Student | 0.512 |  |  |  |  | |
| Student\|Lesson type (SG) | 0.716 |  |  |  |  | |
| Student\|Lesson type (EG) | 1.08 |  |  |  |  | |
| Topic | 0.836 |  |  |  |  | |
