# Supplementary material for: The effect of using games in teaching conservation
Source: PeerJ. 2018 Apr 30;6:e4509. doi: 10.7717/peerj.4509 (PMC5936071; doi:10.7717/peerj.4509)
Supplement: Supplemental Information 12 — 70 questions issued to participants before the course. [file peerj-06-4509-s012.pdf]

Personality test

On this form, there are 70 phrases describing people's behaviors. Please use the rating scale below to describe how accurately each statement describes you. Describe yourself as you generally are now, not as you wish to be in the future. Describe yourself as you honestly see yourself, in relation to other people you know of the same sex as you are, and roughly your same age.

* Required

1. **Please write your ID code that we have given in the email.** *

2. **I am the life of the party.** *
   Check all that apply.
   - [ ] disagree
   - [ ] slightly disagree
   - [ ] neutral
   - [ ] slightly agree
   - [ ] agree

3. **I feel little concern for others.** *
   Check all that apply.
   - [ ] disagree
   - [ ] slightly disagree
   - [ ] neutral
   - [ ] slightly agree
   - [ ] agree

4. **I am always prepared.** *
   Check all that apply.
   - [ ] disagree
   - [ ] slightly disagree
   - [ ] neutral
   - [ ] slightly agree
   - [ ] agree

5. **I get stressed out easily.** *
   Check all that apply.
   - [ ] disagree
   - [ ] slightly disagree
   - [ ] neutral
   - [ ] slightly agree
   - [ ] agree

6. **I have a rich vocabulary.** *
Check all that apply.

- [ ] disagree
- [ ] slightly disagree
- [ ] neutral
- [ ] slightly agree
- [ ] agree

7. **I don't talk a lot.** *
Check all that apply.

- [ ] disagree
- [ ] slightly disagree
- [ ] neutral
- [ ] slightly agree
- [ ] agree

8. **I am interested in people.** *
Check all that apply.

- [ ] disagree
- [ ] slightly disagree
- [ ] neutral
- [ ] slightly agree
- [ ] agree

9. **I leave my belongings around.** *
Check all that apply.

- [ ] disagree
- [ ] slightly disagree
- [ ] neutral
- [ ] slightly agree
- [ ] agree

10. **I am relaxed most of the time.** *
Check all that apply.

- [ ] disagree
- [ ] slightly disagree
- [ ] neutral
- [ ] slightly agree
- [ ] agree

11. **I have difficulty understanding abstract ideas.** *

Check all that apply.

- [ ] disagree
- [ ] slightly disagree
- [ ] neutral
- [ ] slightly agree
- [ ] agree

12. **I feel comfortable around people.** *

Check all that apply.

- [ ] disagree
- [ ] slightly disagree
- [ ] neutral
- [ ] slightly agree
- [ ] agree

13. **I insult people.** *

Check all that apply.

- [ ] disagree
- [ ] slightly disagree
- [ ] neutral
- [ ] slightly agree
- [ ] agree

14. **I pay attention to details.** *

Check all that apply.

- [ ] disagree
- [ ] slightly disagree
- [ ] neutral
- [ ] slightly agree
- [ ] agree

15. **I worry about things.** *

Check all that apply.

- [ ] disagree
- [ ] slightly disagree
- [ ] neutral
- [ ] slightly agree
- [ ] agree

16. **I have a vivid imagination.** *
Check all that apply.

- [ ] disagree
- [ ] slightly disagree
- [ ] neutral
- [ ] slightly agree
- [ ] agree

17. **I keep in the background.** *
Check all that apply.

- [ ] disagree
- [ ] slightly disagree
- [ ] neutral
- [ ] slightly agree
- [ ] agree

18. **I sympathize with others' feelings.** *
Check all that apply.

- [ ] disagree
- [ ] slightly disagree
- [ ] neutral
- [ ] slightly agree
- [ ] agree

19. **I make a mess of things.** *
Check all that apply.

- [ ] disagree
- [ ] slightly disagree
- [ ] neutral
- [ ] slightly agree
- [ ] agree

20. **I seldom feel blue.** *
Check all that apply.

- [ ] disagree
- [ ] slightly disagree
- [ ] neutral
- [ ] slightly agree
- [ ] agree

21. **I am not interested in abstract ideas.** *
Check all that apply.

- [ ] disagree
- [ ] slightly disagree
- [ ] neutral
- [ ] slightly agree
- [ ] agree

22. **I start conversations.** *
Check all that apply.

- [ ] disagree
- [ ] slightly disagree
- [ ] neutral
- [ ] slightly agree
- [ ] agree

23. **I am not interested in other people's problems.** *
Check all that apply.

- [ ] disagree
- [ ] slightly disagree
- [ ] neutral
- [ ] slightly agree
- [ ] agree

24. **I get chores done right away.** *
Check all that apply.

- [ ] disagree
- [ ] slightly disagree
- [ ] neutral
- [ ] slightly agree
- [ ] agree

25. **I am easily disturbed.** *
Check all that apply.

- [ ] disagree
- [ ] slightly disagree
- [ ] neutral
- [ ] slightly agree
- [ ] agree

26. **I have excellent ideas.** *

Check all that apply.

- [ ] disagree
- [ ] slightly disagree
- [ ] neutral
- [ ] slightly agree
- [ ] agree

27. **I have little to say.** *

Check all that apply.

- [ ] disagree
- [ ] slightly disagree
- [ ] neutral
- [ ] slightly agree
- [ ] agree

28. **I have a soft heart.** *

Check all that apply.

- [ ] disagree
- [ ] slightly disagree
- [ ] neutral
- [ ] slightly agree
- [ ] agree

29. **I often forget to put things back in their proper place.** *

Check all that apply.

- [ ] disagree
- [ ] slightly disagree
- [ ] neutral
- [ ] slightly agree
- [ ] agree

30. **I get upset easily.** *

Check all that apply.

- [ ] disagree
- [ ] slightly disagree
- [ ] neutral
- [ ] slightly agree
- [ ] agree

31. **I do not have a good imagination.** *

Check all that apply.

- [ ] disagree
- [ ] slightly disagree
- [ ] neutral
- [ ] slightly agree
- [ ] agree

32. **I talk to a lot of different people at parties.** *

Check all that apply.

- [ ] disagree
- [ ] slightly disagree
- [ ] neutral
- [ ] slightly agree
- [ ] agree

33. **I am not really interested in others.** *

Check all that apply.

- [ ] disagree
- [ ] slightly disagree
- [ ] neutral
- [ ] slightly agree
- [ ] agree

34. **I like order.** *

Check all that apply.

- [ ] disagree
- [ ] slightly disagree
- [ ] neutral
- [ ] slightly agree
- [ ] agree

35. **I change my mood a lot.** *

Check all that apply.

- [ ] disagree
- [ ] slightly disagree
- [ ] neutral
- [ ] slightly agree
- [ ] agree

36. **I am quick to understand things.** *
Check all that apply.

- [ ] disagree
- [ ] slightly disagree
- [ ] neutral
- [ ] slightly agree
- [ ] agree

37. **I don't like to draw attention to myself.** *
Check all that apply.

- [ ] disagree
- [ ] slightly disagree
- [ ] neutral
- [ ] slightly agree
- [ ] agree

38. **I take time out for others.** *
Check all that apply.

- [ ] disagree
- [ ] slightly disagree
- [ ] neutral
- [ ] slightly agree
- [ ] agree

39. **I shirk (avoid) my duties.** *
Check all that apply.

- [ ] disagree
- [ ] slightly disagree
- [ ] neutral
- [ ] slightly agree
- [ ] agree

40. **I have frequent mood swings.** *
Check all that apply.

- [ ] disagree
- [ ] slightly disagree
- [ ] neutral
- [ ] slightly agree
- [ ] agree

41. **I use difficult words.** *

Check all that apply.

- [ ] disagree
- [ ] slightly disagree
- [ ] neutral
- [ ] slightly agree
- [ ] agree

42. **I don't mind being the center of attention.** *

Check all that apply.

- [ ] disagree
- [ ] slightly disagree
- [ ] neutral
- [ ] slightly agree
- [ ] agree

43. **I feel others' emotions.** *

Check all that apply.

- [ ] disagree
- [ ] slightly disagree
- [ ] neutral
- [ ] slightly agree
- [ ] agree

44. **I follow a schedule.** *

Check all that apply.

- [ ] disagree
- [ ] slightly disagree
- [ ] neutral
- [ ] slightly agree
- [ ] agree

45. **I get irritated easily.** *

Check all that apply.

- [ ] disagree
- [ ] slightly disagree
- [ ] neutral
- [ ] slightly agree
- [ ] agree

46. **I spend time reflecting on things.** *

Check all that apply.

- [ ] disagree
- [ ] slightly disagree
- [ ] neutral
- [ ] slightly agree
- [ ] agree

47. **I am quiet around strangers.** *

Check all that apply.

- [ ] disagree
- [ ] slightly disagree
- [ ] neutral
- [ ] slightly agree
- [ ] agree

48. **I make people feel at ease.** *

Check all that apply.

- [ ] disagree
- [ ] slightly disagree
- [ ] neutral
- [ ] slightly agree
- [ ] agree

49. **I am exacting (making great demands on own's skill) in my work.** *

Check all that apply.

- [ ] disagree
- [ ] slightly disagree
- [ ] neutral
- [ ] slightly agree
- [ ] agree

50. **I often feel blue.** *

Check all that apply.

- [ ] disagree
- [ ] slightly disagree
- [ ] neutral
- [ ] slightly agree
- [ ] agree

51. **I am full of ideas.** *

Check all that apply.

- [ ] disagree
- [ ] slightly disagree
- [ ] neutral
- [ ] slightly agree
- [ ] agree

Powered by
Google Forms
