# Supplementary material for: The effect of using games in teaching conservation
Source: PeerJ. 2018 Apr 30;6:e4509. doi: 10.7717/peerj.4509 (PMC5936071; doi:10.7717/peerj.4509)
Supplement: Supplemental Information 13 — 120 questions issued to participants before the course. [file peerj-06-4509-s013.pdf]

Learning style survey

This form describes people's learning behaviour. Please use the rating scale below to describe how accurately each statement describes you. Describe yourself as you generally are now, not as you wish to be in the future.Describe yourself as you honestly see yourself.

* Required

1. **Please fill in your ID code given to you via email.** *

2. **1. I work through a chapter in a textbook item by item and I study each part separately.** *

   Check all that apply.

   - [ ] disagree
   - [ ] slightly disagree
   - [ ] neutral
   - [ ] slightly agree
   - [ ] agree

3. **2. I repeat the main parts of the subject matter until I know them by heart.hrough a chapter in a textbook item by item and I study each part separately.** *

   Check all that apply.

   - [ ] disagree
   - [ ] slightly disagree
   - [ ] neutral
   - [ ] slightly agree
   - [ ] agree

4. **3. I use what I learn from a course in my activities outside my studies.** *

   Check all that apply.

   - [ ] disagree
   - [ ] slightly disagree
   - [ ] neutral
   - [ ] slightly agree
   - [ ] agree

5. **4. If a textbook contains questions or assignments, I work them out completely as soon as I come across them while studying.** *

Check all that apply.

- [ ] disagree
- [ ] slightly disagree
- [ ] neutral
- [ ] slightly agree
- [ ] agree

6. **5. I study all the subject matter in the same way.** *

Check all that apply.

- [ ] disagree
- [ ] slightly disagree
- [ ] neutral
- [ ] slightly agree
- [ ] agree

7. **6. I try to combine the subjects that are dealt with separately in a course into one whole.** *

Check all that apply.

- [ ] disagree
- [ ] slightly disagree
- [ ] neutral
- [ ] slightly agree
- [ ] agree

8. **7. I memorize lists of characteristics of a certain phenomenon.** *

Check all that apply.

- [ ] disagree
- [ ] slightly disagree
- [ ] neutral
- [ ] slightly agree
- [ ] agree

9. **8. I realize that it is not clear to me what I have to remember and what I do not have to remember.** *

Check all that apply.

- [ ] disagree
- [ ] slightly disagree
- [ ] neutral
- [ ] slightly agree
- [ ] agree

10. **9. I make a list of the most important facts and learn them by heart.** *

Check all that apply.

- [ ] disagree
- [ ] slightly disagree
- [ ] neutral
- [ ] slightly agree
- [ ] agree

11. **10. I try to discover the similarities and differences between the theories that are dealt with in a course.** *

Check all that apply.

- [ ] disagree
- [ ] slightly disagree
- [ ] neutral
- [ ] slightly agree
- [ ] agree

12. **11. I experience the introductions, objectives, instructions, assignments and test items given by the teacher as indispensable guidelines for my studies.** *

Check all that apply.

- [ ] disagree
- [ ] slightly disagree
- [ ] neutral
- [ ] slightly agree
- [ ] agree

13. **12. I test my learning progress solely by completing the questions, tasks and exercises provided by the teacher or the textbook.** *

Check all that apply.

- [ ] disagree
- [ ] slightly disagree
- [ ] neutral
- [ ] slightly agree
- [ ] agree

14. **13. I relate specific facts to the main issue in a chapter or article.** *

Check all that apply.

- [ ] disagree
- [ ] slightly disagree
- [ ] neutral
- [ ] slightly agree
- [ ] agree

15. **14. I try to interpret events in everyday reality with the help of the knowledge I have acquired in a course.** *
Check all that apply.

- [ ] disagree
- [ ] slightly disagree
- [ ] neutral
- [ ] slightly agree
- [ ] agree

16. **15. I notice that I have trouble processing a large amount of subject matter.** *
Check all that apply.

- [ ] disagree
- [ ] slightly disagree
- [ ] neutral
- [ ] slightly agree
- [ ] agree

17. **16. In addition to the syllabus, I study other literature related to the content of the course.** *
Check all that apply.

- [ ] disagree
- [ ] slightly disagree
- [ ] neutral
- [ ] slightly agree
- [ ] agree

18. **17. I analyse the separate components of a theory step by step.** *
Check all that apply.

- [ ] disagree
- [ ] slightly disagree
- [ ] neutral
- [ ] slightly agree
- [ ] agree

19. **18. I learn everything exactly as I find it in the textbooks.** *
Check all that apply.

- [ ] disagree
- [ ] slightly disagree
- [ ] neutral
- [ ] slightly agree
- [ ] agree

20. **19. I try to relate new subject matter to knowledge I already have about the topic concerned.** *

Check all that apply.

- [ ] disagree
- [ ] slightly disagree
- [ ] neutral
- [ ] slightly agree
- [ ] agree

21. **20. I notice that it is difficult for me to determine whether I have mastered the subject matter sufficiently.** *

Check all that apply.

- [ ] disagree
- [ ] slightly disagree
- [ ] neutral
- [ ] slightly agree
- [ ] agree

22. **21. To test my learning progress when I have studied a textbook, I try to formulate the main points in my own words.** *

Check all that apply.

- [ ] disagree
- [ ] slightly disagree
- [ ] neutral
- [ ] slightly agree
- [ ] agree

23. **22. I pay particular attention to those parts of a course that have practical utility.** *

Check all that apply.

- [ ] disagree
- [ ] slightly disagree
- [ ] neutral
- [ ] slightly agree
- [ ] agree

24. **23. I do not proceed to a subsequent chapter until I have mastered the current chapter in detail.** *

Check all that apply.

- [ ] disagree
- [ ] slightly disagree
- [ ] neutral
- [ ] slightly agree
- [ ] agree

25. **24. When I start reading a new chapter or article, I first think about the best way to study it.** *

Check all that apply.

- [ ] disagree
- [ ] slightly disagree
- [ ] neutral
- [ ] slightly agree
- [ ] agree

26. **25. I try to see the connection between the topics discussed in different chapters of a textbook.** *

Check all that apply.

- [ ] disagree
- [ ] slightly disagree
- [ ] neutral
- [ ] slightly agree
- [ ] agree

27. **26. I memorize definitions as literally as possible.** *

Check all that apply.

- [ ] disagree
- [ ] slightly disagree
- [ ] neutral
- [ ] slightly agree
- [ ] agree

28. **27. I realize that the objectives of the course are too general for me to offer any support.** *

Check all that apply.

- [ ] disagree
- [ ] slightly disagree
- [ ] neutral
- [ ] slightly agree
- [ ] agree

29. **28. I do more than I am expected to do in a course.** *

Check all that apply.

- [ ] disagree
- [ ] slightly disagree
- [ ] neutral
- [ ] slightly agree
- [ ] agree

30. **29. I compare my view of a course topic with the views of the authors of the textbook used in that course.** *

Check all that apply.

- [ ] disagree
- [ ] slightly disagree
- [ ] neutral
- [ ] slightly agree
- [ ] agree

31. **30. If I am able to give a good answer to the questions posed in the textbook or by the teacher, I decide that I have a good command of the subject matter.** *

Check all that apply.

- [ ] disagree
- [ ] slightly disagree
- [ ] neutral
- [ ] slightly agree
- [ ] agree

32. **31. When I have difficulty grasping a particular piece of subject matter, I try to analyse why it is difficult for me.** *

Check all that apply.

- [ ] disagree
- [ ] slightly disagree
- [ ] neutral
- [ ] slightly agree
- [ ] agree

33. **32. I study according to the instructions given in the study materials or provided by the teacher.** *

Check all that apply.

- [ ] disagree
- [ ] slightly disagree
- [ ] neutral
- [ ] slightly agree
- [ ] agree

34. **33. I memorize the meaning of every concept that is unfamiliar to me.** *

Check all that apply.

- [ ] disagree
- [ ] slightly disagree
- [ ] neutral
- [ ] slightly agree
- [ ] agree

35. **34. I try to construct an overall picture of a course for myself.** *

Check all that apply.

- [ ] disagree
- [ ] slightly disagree
- [ ] neutral
- [ ] slightly agree
- [ ] agree

36. **35. I compare the conclusions drawn in different chapters.** *

Check all that apply.

- [ ] disagree
- [ ] slightly disagree
- [ ] neutral
- [ ] slightly agree
- [ ] agree

37. **36. To test my learning progress, I try to answer questions about the subject matter which I make up myself.** *

Check all that apply.

- [ ] disagree
- [ ] slightly disagree
- [ ] neutral
- [ ] slightly agree
- [ ] agree

38. **37. I notice that the study instructions that are given are not very clear to me.** *

Check all that apply.

- [ ] disagree
- [ ] slightly disagree
- [ ] neutral
- [ ] slightly agree
- [ ] agree

39. **38. I study the subject matter in the same sequence as it is dealt with in the course.** *

Check all that apply.

- [ ] disagree
- [ ] slightly disagree
- [ ] neutral
- [ ] slightly agree
- [ ] agree

40. **39. I check whether the conclusions drawn by the authors of a textbook follow the facts on which they are based logically.** *

Check all that apply.

- [ ] disagree
- [ ] slightly disagree
- [ ] neutral
- [ ] slightly agree
- [ ] agree

41. **40. I study details thoroughly.** *

Check all that apply.

- [ ] disagree
- [ ] slightly disagree
- [ ] neutral
- [ ] slightly agree
- [ ] agree

42. **41. I realize that I miss someone to fall back on in case of difficulties.** *

Check all that apply.

- [ ] disagree
- [ ] slightly disagree
- [ ] neutral
- [ ] slightly agree
- [ ] agree

43. **42. I add something to the subject matter from other sources.** *

Check all that apply.

- [ ] disagree
- [ ] slightly disagree
- [ ] neutral
- [ ] slightly agree
- [ ] agree

44. **43. I draw my own conclusions on the basis of the data that are presented in a course.** *

Check all that apply.

- [ ] disagree
- [ ] slightly disagree
- [ ] neutral
- [ ] slightly agree
- [ ] agree

45. **44. When doing assignments, I train myself thoroughly in applying the methods dealt with in a course.** *
Check all that apply.

- [ ] disagree
- [ ] slightly disagree
- [ ] neutral
- [ ] slightly agree
- [ ] agree

46. **45. I analyse the successive steps in an argumentation one by one.** *
Check all that apply.

- [ ] disagree
- [ ] slightly disagree
- [ ] neutral
- [ ] slightly agree
- [ ] agree

47. **46. To test whether I have mastered the subject matter, I try to think up other examples and problems besides the ones given in the study materials or by the teacher.** *
Check all that apply.

- [ ] disagree
- [ ] slightly disagree
- [ ] neutral
- [ ] slightly agree
- [ ] agree

48. **47. I use the instructions and the course objectives given by the teacher to know exactly what to do.** *
Check all that apply.

- [ ] disagree
- [ ] slightly disagree
- [ ] neutral
- [ ] slightly agree
- [ ] agree

49. **48. With the help of the theories presented in a course, I devise solutions to practical problems.** *
Check all that apply.

- [ ] disagree
- [ ] slightly disagree
- [ ] neutral
- [ ] slightly agree
- [ ] agree

50. **49. I try to be critical of the interpretations of experts.** *

Check all that apply.

- [ ] disagree
- [ ] slightly disagree
- [ ] neutral
- [ ] slightly agree
- [ ] agree

51. **50. To test my own progress, I try to describe the content of a paragraph in my own words.** *

Check all that apply.

- [ ] disagree
- [ ] slightly disagree
- [ ] neutral
- [ ] slightly agree
- [ ] agree

52. **51. When I am studying, I also pursue learning goals that have not been set by the teacher but by myself.** *

Check all that apply.

- [ ] disagree
- [ ] slightly disagree
- [ ] neutral
- [ ] slightly agree
- [ ] agree

53. **52. When I am studying a topic, I think of cases I know from my own experience that are connected to that topic.** *

Check all that apply.

- [ ] disagree
- [ ] slightly disagree
- [ ] neutral
- [ ] slightly agree
- [ ] agree

54. **53. I pay particular attention to facts, concepts and problem solving methods in a course.** *

Check all that apply.

- [ ] disagree
- [ ] slightly disagree
- [ ] neutral
- [ ] slightly agree
- [ ] agree

55. **54. If I do not understand a study text well, I try to find other literature about the subject concerned.** *

Check all that apply.

- [ ] disagree
- [ ] slightly disagree
- [ ] neutral
- [ ] slightly agree
- [ ] agree

56. **55. If I am able to complete all the assignments given in the study materials or by the teacher, I decide that I have a good command of the subject matter.** *

Check all that apply.

- [ ] disagree
- [ ] slightly disagree
- [ ] neutral
- [ ] slightly agree
- [ ] agree

57. **56. When I have a choice, I opt for courses that seem useful to me for my present or future profession.** *

Check all that apply.

- [ ] disagree
- [ ] slightly disagree
- [ ] neutral
- [ ] slightly agree
- [ ] agree

58. **57. I do these studies out of sheer interest in the topics that are dealt with.** *

Check all that apply.

- [ ] disagree
- [ ] slightly disagree
- [ ] neutral
- [ ] slightly agree
- [ ] agree

59. **58. I want to prove to myself that I am capable of doing studies in higher education.** *

Check all that apply.

- [ ] disagree
- [ ] slightly disagree
- [ ] neutral
- [ ] slightly agree
- [ ] agree

60. **59. I doubt whether this is the right subject area for me.** *
Check all that apply.

- [ ] disagree
- [ ] slightly disagree
- [ ] neutral
- [ ] slightly agree
- [ ] agree

61. **60. I aim at attaining high levels of study achievements.** *
Check all that apply.

- [ ] disagree
- [ ] slightly disagree
- [ ] neutral
- [ ] slightly agree
- [ ] agree

62. **61. I want to show others that I am capable of successfully doing a higher education programme.** *
Check all that apply.

- [ ] disagree
- [ ] slightly disagree
- [ ] neutral
- [ ] slightly agree
- [ ] agree

63. **62. I have chosen this subject area, because it prepares me for the type of work I am highly interested in.** *
Check all that apply.

- [ ] disagree
- [ ] slightly disagree
- [ ] neutral
- [ ] slightly agree
- [ ] agree

64. **63. The main goal I pursue in my studies is to pass exams.** *
Check all that apply.

- [ ] disagree
- [ ] slightly disagree
- [ ] neutral
- [ ] slightly agree
- [ ] agree

65. **64. I view the choice I have made to enrol in higher education as a challenge.** *
Check all that apply.

- [ ] disagree
- [ ] slightly disagree
- [ ] neutral
- [ ] slightly agree
- [ ] agree

66. **65. The only aim of my studies is to enrich myself.** *
Check all that apply.

- [ ] disagree
- [ ] slightly disagree
- [ ] neutral
- [ ] slightly agree
- [ ] agree

67. **66. I have little confidence in my study capacities.** *
Check all that apply.

- [ ] disagree
- [ ] slightly disagree
- [ ] neutral
- [ ] slightly agree
- [ ] agree

68. **67. For the kind of work I would like to do, I need to have studied in higher education.** *
Check all that apply.

- [ ] disagree
- [ ] slightly disagree
- [ ] neutral
- [ ] slightly agree
- [ ] agree

69. **68. What I want in these studies is to earn credits for a diploma.** *
Check all that apply.

- [ ] disagree
- [ ] slightly disagree
- [ ] neutral
- [ ] slightly agree
- [ ] agree

70. **69. I see these studies as sheer relaxation.** *

Check all that apply.

- [ ] disagree
- [ ] slightly disagree
- [ ] neutral
- [ ] slightly agree
- [ ] agree

71. **70. I study above all to pass the exam.** *

Check all that apply.

- [ ] disagree
- [ ] slightly disagree
- [ ] neutral
- [ ] slightly agree
- [ ] agree

72. **71. The main goal I pursue in my studies is to prepare myself for a profession.** *

Check all that apply.

- [ ] disagree
- [ ] slightly disagree
- [ ] neutral
- [ ] slightly agree
- [ ] agree

73. **72. I want to discover my own qualities, the things I am capable and incapable of.** *

Check all that apply.

- [ ] disagree
- [ ] slightly disagree
- [ ] neutral
- [ ] slightly agree
- [ ] agree

74. **73. What I want to acquire above all through my studies is professional skill.** *

Check all that apply.

- [ ] disagree
- [ ] slightly disagree
- [ ] neutral
- [ ] slightly agree
- [ ] agree

75. **74. When I have a choice, I opt for courses that suit my personal interests.** *

Check all that apply.

- [ ] disagree
- [ ] slightly disagree
- [ ] neutral
- [ ] slightly agree
- [ ] agree

76. **75. I wonder whether these studies are worth all the effort.** *

Check all that apply.

- [ ] disagree
- [ ] slightly disagree
- [ ] neutral
- [ ] slightly agree
- [ ] agree

77. **76. I doubt whether this type of education is the right type of education for me.** *

Check all that apply.

- [ ] disagree
- [ ] slightly disagree
- [ ] neutral
- [ ] slightly agree
- [ ] agree

78. **77. I want to test myself to see whether I am capable of doing studies in higher education.** *

Check all that apply.

- [ ] disagree
- [ ] slightly disagree
- [ ] neutral
- [ ] slightly agree
- [ ] agree

79. **78. I do these studies because I like to learn and to study.** *

Check all that apply.

- [ ] disagree
- [ ] slightly disagree
- [ ] neutral
- [ ] slightly agree
- [ ] agree

80. **79. I am afraid these studies are too demanding for me.** *

Check all that apply.

- [ ] disagree
- [ ] slightly disagree
- [ ] neutral
- [ ] slightly agree
- [ ] agree

81. **80. To me, written proof of having passed an exam represents something of value in itself.** *

Check all that apply.

- [ ] disagree
- [ ] slightly disagree
- [ ] neutral
- [ ] slightly agree
- [ ] agree

82. **81. The things I learn have to be useful for solving practical problems.** *

Check all that apply.

- [ ] disagree
- [ ] slightly disagree
- [ ] neutral
- [ ] slightly agree
- [ ] agree

83. **82. I like to be given precise instructions as to how to go about solving a task or doing an assignment.** *

Check all that apply.

- [ ] disagree
- [ ] slightly disagree
- [ ] neutral
- [ ] slightly agree
- [ ] agree

84. **83. The teacher should motivate and encourage me.** *

Check all that apply.

- [ ] disagree
- [ ] slightly disagree
- [ ] neutral
- [ ] slightly agree
- [ ] agree

85. **84. When I prepare myself for an exam, I prefer to do so together with other students.** *
Check all that apply.

- [ ] disagree
- [ ] slightly disagree
- [ ] neutral
- [ ] slightly agree
- [ ] agree

86. **85. To me, learning means trying to approach a problem from many different angles, including aspects that were previously unknown to me.** *
Check all that apply.

- [ ] disagree
- [ ] slightly disagree
- [ ] neutral
- [ ] slightly agree
- [ ] agree

87. **86. To me, learning is making sure that I can reproduce the facts presented in a course.** *
Check all that apply.

- [ ] disagree
- [ ] slightly disagree
- [ ] neutral
- [ ] slightly agree
- [ ] agree

88. **87. The teacher should inspire me to work out how the course material relates to reality.** *
Check all that apply.

- [ ] disagree
- [ ] slightly disagree
- [ ] neutral
- [ ] slightly agree
- [ ] agree

89. **88. I should look for relationships within the subject matter of my own accord.** *
Check all that apply.

- [ ] disagree
- [ ] slightly disagree
- [ ] neutral
- [ ] slightly agree
- [ ] agree

90. **89. I like to be encouraged by other students to process the study materials at a particular pace.** *

Check all that apply.

- [ ] disagree
- [ ] slightly disagree
- [ ] neutral
- [ ] slightly agree
- [ ] agree

91. **90. I should try myself to apply the theories dealt with in a course to practical situations.** *

Check all that apply.

- [ ] disagree
- [ ] slightly disagree
- [ ] neutral
- [ ] slightly agree
- [ ] agree

92. **91. The teacher should encourage me to combine the separate components of a course into a whole.** *

Check all that apply.

- [ ] disagree
- [ ] slightly disagree
- [ ] neutral
- [ ] slightly agree
- [ ] agree

93. **92. If I have difficulty understanding a particular topic, I should consult other books of my own accord.** *

Check all that apply.

- [ ] disagree
- [ ] slightly disagree
- [ ] neutral
- [ ] slightly agree
- [ ] agree

94. **93. I prefer to do assignments together with other students.** *

Check all that apply.

- [ ] disagree
- [ ] slightly disagree
- [ ] neutral
- [ ] slightly agree
- [ ] agree

95. **94. The teacher should explain clearly what is important and what is less important for me to know.** *

Check all that apply.

- [ ] disagree
- [ ] slightly disagree
- [ ] neutral
- [ ] slightly agree
- [ ] agree

96. **95. I have a preference for courses in which a lot of practical applications of the theoretical parts are given.** *

Check all that apply.

- [ ] disagree
- [ ] slightly disagree
- [ ] neutral
- [ ] slightly agree
- [ ] agree

97. **96. In order to learn, I have to summarize in my own words what the subject matter means.** *

Check all that apply.

- [ ] disagree
- [ ] slightly disagree
- [ ] neutral
- [ ] slightly agree
- [ ] agree

98. **97. When I have difficulty understanding something, the teacher should encourage me to find a solution by myself.** *

Check all that apply.

- [ ] disagree
- [ ] slightly disagree
- [ ] neutral
- [ ] slightly agree
- [ ] agree

99. **98. I think I can not just rely on the books recommended by the syllabus, so I have to try to discover myself what else has been written about a particular course topic.** *

Check all that apply.

- [ ] disagree
- [ ] slightly disagree
- [ ] neutral
- [ ] slightly agree
- [ ] agree

100. **99. I think it is important to check with other students to see whether I have sufficiently understood the subject matter.** *
Check all that apply.

- [ ] disagree
- [ ] slightly disagree
- [ ] neutral
- [ ] slightly agree
- [ ] agree

101. **100. I should memorize definitions and other facts on my own.** *
Check all that apply.

- [ ] disagree
- [ ] slightly disagree
- [ ] neutral
- [ ] slightly agree
- [ ] agree

102. **101. When I have difficulties, the teacher should encourage me to find out for myself what causes them.** *
Check all that apply.

- [ ] disagree
- [ ] slightly disagree
- [ ] neutral
- [ ] slightly agree
- [ ] agree

103. **102. To me, learning means acquiring knowledge that I can use in everyday life.** *
Check all that apply.

- [ ] disagree
- [ ] slightly disagree
- [ ] neutral
- [ ] slightly agree
- [ ] agree

104. **103. Good teaching includes giving a lot of questions and exercises to test whether I have mastered the subject matter.** *
Check all that apply.

- [ ] disagree
- [ ] slightly disagree
- [ ] neutral
- [ ] slightly agree
- [ ] agree

105. **104. To test my own learning progress, I should try to answer questions about the subject matter which I make up myself.** *

Check all that apply.

- [ ] disagree
- [ ] slightly disagree
- [ ] neutral
- [ ] slightly agree
- [ ] agree

106. **105. The teacher should encourage me to compare the various theories that are dealt with in a course.** *

Check all that apply.

- [ ] disagree
- [ ] slightly disagree
- [ ] neutral
- [ ] slightly agree
- [ ] agree

107. **106. I should repeat the subject matter on my own until I know it sufficiently.** *

Check all that apply.

- [ ] disagree
- [ ] slightly disagree
- [ ] neutral
- [ ] slightly agree
- [ ] agree

108. **107. I prefer a type of instruction in which I am told exactly what I need to know for an exam.** *

Check all that apply.

- [ ] disagree
- [ ] slightly disagree
- [ ] neutral
- [ ] slightly agree
- [ ] agree

109. **108. To me, learning is providing myself with information that I can use immediately or in the longer term.** *

Check all that apply.

- [ ] disagree
- [ ] slightly disagree
- [ ] neutral
- [ ] slightly agree
- [ ] agree

110. **109. I consider it important to be advised by other students as to how to approach my studies.** *
Check all that apply.

- [ ] disagree
- [ ] slightly disagree
- [ ] neutral
- [ ] slightly agree
- [ ] agree

111. **110. The teacher should encourage me to check myself whether I have mastered the subject matter.** *
Check all that apply.

- [ ] disagree
- [ ] slightly disagree
- [ ] neutral
- [ ] slightly agree
- [ ] agree

112. **111. When I have difficulty understanding particular topics, I prefer to ask other students for help.** *
Check all that apply.

- [ ] disagree
- [ ] slightly disagree
- [ ] neutral
- [ ] slightly agree
- [ ] agree

113. **112. To me, learning means: trying to remember the subject matter I am given.** *
Check all that apply.

- [ ] disagree
- [ ] slightly disagree
- [ ] neutral
- [ ] slightly agree
- [ ] agree

114. **113. The teacher should give trial tests to enable me to check whether I have mastered all of the subject matter.** *
Check all that apply.

- [ ] disagree
- [ ] slightly disagree
- [ ] neutral
- [ ] slightly agree
- [ ] agree

115. **114. To me, learning means acquiring knowledge and skills that I can later apply in practice.** *

Check all that apply.

- [ ] disagree
- [ ] slightly disagree
- [ ] neutral
- [ ] slightly agree
- [ ] agree

116. **115. I consider it important to discuss the subject matter with other students.** *

Check all that apply.

- [ ] disagree
- [ ] slightly disagree
- [ ] neutral
- [ ] slightly agree
- [ ] agree

117. **116. I think good teaching is teaching that includes some preparation on my own part.** *

Check all that apply.

- [ ] disagree
- [ ] slightly disagree
- [ ] neutral
- [ ] slightly agree
- [ ] agree

118. **117. I should try to think up examples with the study materials of my own accord.** *

Check all that apply.

- [ ] disagree
- [ ] slightly disagree
- [ ] neutral
- [ ] slightly agree
- [ ] agree

119. **118. The teacher should encourage me to reflect on the way I study and how to develop my way of studying.** *

Check all that apply.

- [ ] disagree
- [ ] slightly disagree
- [ ] neutral
- [ ] slightly agree
- [ ] agree

120. **119. In order to check whether I have mastered the subject matter, I should try to describe the main points in my own words.** *

Check all that apply.

- [ ] disagree
- [ ] slightly disagree
- [ ] neutral
- [ ] slightly agree
- [ ] agree

121. **120. I have a need to work together with other students in my studies.** *

Check all that apply.

- [ ] disagree
- [ ] slightly disagree
- [ ] neutral
- [ ] slightly agree
- [ ] agree

Powered by
Google Forms
