# Supplementary material for: The effect of using games in teaching conservation
Source: PeerJ. 2018 Apr 30;6:e4509. doi: 10.7717/peerj.4509 (PMC5936071; doi:10.7717/peerj.4509)
Supplement: Supplemental Information 14 — DI – Didactic Instruction; SG – Supplemental Game; EG – Experiential Game. Definition of other explanatory variables [reference level for discrete variables]: BeforeAfter – Before or After lesson; Year – year of course [2015]; Course – Diploma in Oxford or Wildlife Conservation Course in Malaysia [Diploma]; Topic – topic of lesson. The second column shows either (i) the estimate of the slope for a continuous fixed variable, (ii) the estimate of the difference in mean from the reference level for a discrete fixed variable, or (iii) the variance of a random variable. Significant variables are highlighted in bold. [file peerj-06-4509-s014.docx]

Supplementary Table S7. Table of learning results as analysed using Generalised Linear Mixed Models with Binomial error distribution on standardised quiz scores. DI – Didactic Instruction; SG – Supplemental Game; EG – Experiential Game. Definition of other explanatory variables [reference level for discrete variables]: BeforeAfter – Before or After lesson; Year – year of course [2015]; Course – Diploma in Oxford or Wildlife Conservation Course in Malaysia [Diploma]; Topic – topic of lesson. The second column shows either (i) the estimate of the slope for a continuous fixed variable, (ii) the estimate of the difference in mean from the reference level for a discrete fixed variable, or (iii) the variance of a random variable. Significant variables are highlighted in bold.

|  | Estimate (fixed) / Variance (random) | SE | df | χ^2^ | *p* |
| --- | --- | --- | --- | --- | --- |
| All question types | | | | | |
| Both years | | | | | |
| (Intercept) | 67.271 | 151.62 |  |  |  |
| BeforeAfter*Lesson type (SG) | 0.000 | 0.000 | 2 | 0.418 | 0.811 |
| BeforeAfter*Lesson type (EG) | 0.000 | 0.000 |  |  |  |
| BeforeAfter | 0.010 | 0.053 | 1 | 0.064 | 0.800 |
| Lesson type (SG) | 0.082 | 0.068 | 2 | 2.109 | 0.348 |
| Lesson type (EG) | 0.139 | 0.065 |  |  |  |
| Year | -0.033 | 0.075 | 1 | 0.320 | 0.571 |
| Course | 0.006 | 0.084 | 1 | 3.441 | 0.064 |
| Student | 0.183 |  |  |  |  |
| Student\|Lesson type (SG) | 0.000 |  |  |  |  |
| Student\|Lesson type (EG) | 0.000 |  |  |  |  |
| Subject | 0.000 |  |  |  |  |
|  |  |  |  |  |  |
| Year 2015 | | | | | |
| (Intercept) | 0.057 | 0.079 |  |  |  |
| BeforeAfter*Lesson type (SG) | 0.059 | 0.072 | 2 | 1.611 | 0.447 |
| BeforeAfter*Lesson type (EG) | 0.091 | 0.071 |  |  |  |
| BeforeAfter | -0.066 | 0.055 | 1 | 0.165 | 0.684 |
| Lesson type (SG) | -0.016 | 0.052 | 2 | 1.194 | 0.551 |
| Lesson type (EG) | -0.071 | 0.051 |  |  |  |
| Course | -0.004 | 0.082 | 1 | 0.002 | 0.969 |
| Student | 0.182 |  |  |  |  |
| Student\|Lesson type (SG) | 0.023 |  |  |  |  |
| Student\|Lesson type (EG) | 0.000 |  |  |  |  |
| Subject | 0.000 |  |  |  |  |
|  |  |  |  |  |  |
| Year 2016 | | | | | |
| (Intercept) | -0.057 | 0.064 |  |  |  |
| BeforeAfterLT (Before)*Lesson type (EG) | -0.017 | 0.068 | 4 | 0.290 | 0.990 |
| BeforeAfterLT (Before)*Lesson type (SG) | -0.017 | 0.093 |  |  |  |
| BeforeAfterLT (LT)*Lesson type (EG) | -0.012 | 0.069 |  |  |  |
| BeforeAfterLT (LT)*Lesson type (SG) | 0.024 | 0.092 |  |  |  |
| BeforeAfterLT (Before) | 0.013 | 0.049 | 2 | 1.548 | 0.461 |
| BeforeAfterLT (LT) | 0.037 | 0.049 |  |  |  |
| Lesson type (SG) | -0.011 | 0.065 | 2 | 1.682 | 0.431 |
| Lesson type (EG) | 0.041 | 0.048 |  |  |  |
| Course | 0.001 | 0.069 | 1 | 0.000 | 1.000 |
| Student | 0.149 |  |  |  |  |
| Student\|Lesson type (SG) | 0.000 |  |  |  |  |
| Student\|Lesson type (EG) | 0.000 |  |  |  |  |
| Subject | 0.000 |  |  |  |  |
|  |  |  |  |  |  |
| Reproduction questions | | | | | |
| Both years | | | | | |
| (Intercept) | 0.253 | 0.210 |  |  |  |
| BeforeAfter*Lesson type (SG) | 0.145 | 0.074 | 2 | 3.888 | 0.143 |
| BeforeAfter*Lesson type (EG) | 0.090 | 0.077 |  |  |  |
| BeforeAfter | -0.081 | 0.054 | 1 | 0.000 | 0.999 |
| Lesson type (SG) | -0.029 | 0.053 | 2 | 0.182 | 0.913 |
| Lesson type (EG) | -0.072 | 0.053 |  |  |  |
| Year | -0.017 | 0.061 | 1 | 0.064 | 0.800 |
| Course | 0.000 | 0.066 | 1 | 0.001 | 0.973 |
| Reproduction-directed learning score | -0.004 | 0.004 | 1 | 1.223 | 0.269 |
| Student | 0.189 |  |  |  |  |
| Student\|Lesson type (SG) | 0.000 |  |  |  |  |
| Student\|Lesson type (EG) | 0.000 |  |  |  |  |
| Subject | 0.000 |  |  |  |  |
|  |  |  |  |  |  |
| Year 2015 | | | | | |
| (Intercept) | 0.680 | 0.258 |  |  |  |
| **BeforeAfter*Lesson type (SG)** | **0.203** | **0.102** | **2** | **6.230** | **0.044** |
| **BeforeAfter*Lesson type (EG)** | **0.250** | **0.106** |  |  |  |
| BeforeAfter | -0.178 | 0.078 | 1 | 0.125 | 0.723 |
| Lesson type (SG) | -0.095 | 0.069 | 2 | 5.225 | 0.073 |
| Lesson type (EG) | -0.225 | 0.076 |  |  |  |
| Course | -0.057 | 0.087 | 1 | 0.526 | 0.468 |
| **Reproduction-directed learning score** | **-0.009** | **0.004** | **1** | **4.822** | **0.028** |
| Student | 0.174 |  |  |  |  |
| Student\|Lesson type (SG) | 0.000 |  |  |  |  |
| Student\|Lesson type (EG) | 0.000 |  |  |  |  |
| Subject | 0.000 |  |  |  |  |
|  |  |  |  |  |  |
| Year 2016 | | | | | |
| (Intercept) | -0.319 | 0.342 |  |  |  |
| BeforeAfterLT (Before)*Lesson type (EG) | 0.047 | 0.103 | 4 | 0.830 | 0.934 |
| BeforeAfterLT (Before)*Lesson type (SG) | -0.013 | 0.126 |  |  |  |
| BeforeAfterLT (LT)*Lesson type (EG) | -0.015 | 0.104 |  |  |  |
| BeforeAfterLT (LT)*Lesson type (SG) | 0.028 | 0.124 |  |  |  |
| BeforeAfterLT (Before) | 0.001 | 0.075 | 2 | 0.250 | 0.882 |
| BeforeAfterLT (LT) | 0.021 | 0.074 |  |  |  |
| Lesson type (SG) | -0.007 | 0.088 | 2 | 3.858 | 0.145 |
| Lesson type (EG) | 0.064 | 0.073 |  |  |  |
| Course | 0.004 | 0.096 | 1 | 0.000 | 1.000 |
| Reproduction-directed learning score | 0.021 | 0.046 | 1 | 0.670 | 0.413 |
| Student | 0.199 |  |  |  |  |
| Student\|Lesson type (SG) | 0.000 |  |  |  |  |
| Student\|Lesson type (EG) | 0.000 |  |  |  |  |
| Subject | 0.000 |  |  |  |  |
|  |  |  |  |  |  |
| Meaning questions | | | | | |
| Both years | | | | | |
| (Intercept) | -0.006 | 0.265 |  |  |  |
| BeforeAfter*Lesson type (SG) | -0.035 | 0.076 | 2 | 0.301 | 0.860 |
| BeforeAfter*Lesson type (EG) | -0.044 | 0.091 |  |  |  |
| BeforeAfter | 0.011 | 0.058 | 1 | 0.205 | 0.651 |
| Lesson type (SG) | -0.011 | 0.066 | 2 | 1.072 | 0.585 |
| Lesson type (EG) | -0.022 | 0.054 |  |  |  |
| **Year** | **-0.099** | **0.051** | **1** | **3.859** | **0.049** |
| Course | -0.003 | 0.055 | 1 | 0.004 | 0.949 |
| Meaning-directed learning score | 0.000 | 0.002 | 1 | 0.085 | 0.770 |
| Student | 0.135 |  |  |  |  |
| Student\|Lesson type (SG) | 0.000 |  |  |  |  |
| Student\|Lesson type (EG) | 0.000 |  |  |  |  |
| Subject | 0.000 |  |  |  |  |
|  |  |  |  |  |  |
| Year 2015 | | | | | |
| (Intercept) | -0.328 | 0.431 |  |  |  |
| BeforeAfter*Lesson type (SG) | -0.054 | 0.121 | 2 | 0.243 | 0.885 |
| BeforeAfter*Lesson type (EG) | -0.015 | 0.115 |  |  |  |
| BeforeAfter | 0.000 | 0.093 | 1 | 0.315 | 0.574 |
| Lesson type (SG) | -0.012 | 0.085 | 2 | 0.437 | 0.804 |
| Lesson type (EG) | -0.025 | 0.081 |  |  |  |
| Course | 0.017 | 0.085 | 1 | 0.048 | 0.826 |
| Meaning-directed learning score | 0.003 | 0.003 | 1 | 1.014 | 0.314 |
| Student | 0.145 |  |  |  |  |
| Student\|Lesson type (SG) | 0.000 |  |  |  |  |
| Student\|Lesson type (EG) | 0.000 |  |  |  |  |
| Subject | 0.000 |  |  |  |  |
|  |  |  |  |  |  |
| Year 2016 | | | | | |
| (Intercept) | 0.246 | 0.322 |  |  |  |
| BeforeAfterLT (Before)*Lesson type (EG) | -0.048 | 0.103 | 4 | 0.590 | 0.964 |
| BeforeAfterLT (Before)*Lesson type (SG) | 0.051 | 0.196 |  |  |  |
| BeforeAfterLT (LT)*Lesson type (EG) | 0.014 | 0.104 |  |  |  |
| BeforeAfterLT (LT)*Lesson type (SG) | 0.028 | 0.191 |  |  |  |
| BeforeAfterLT (Before) | 0.016 | 0.075 | 2 | 1.720 | 0.423 |
| BeforeAfterLT (LT) | 0.046 | 0.075 |  |  |  |
| Lesson type (SG) | -0.027 | 0.139 | 2 | 0.605 | 0.739 |
| Lesson type (EG) | -0.021 | 0.074 |  |  |  |
| Course | 0.010 | 0.075 | 1 | 0.028 | 0.868 |
| Meaning-directed learning score | 0.054 | 0.050 | 1 | 0.984 | 0.321 |
| Student | 0.138 |  |  |  |  |
| Student\|Lesson type (SG) | 0.000 |  |  |  |  |
| Student\|Lesson type (EG) | 0.000 |  |  |  |  |
| Subject | 0.000 |  |  |  |  |
|  |  |  |  |  |  |
| Application questions | | | | | |
| Both years | | | | | |
| (Intercept) | -0.413 | 0.423 |  |  |  |
| BeforeAfter*Lesson type (SG) | -0.099 | 0.128 | 2 | 0.782 | 0.676 |
| BeforeAfter*Lesson type (EG) | -0.102 | 0.135 |  |  |  |
| BeforeAfter | 0.078 | 0.094 | 1 | 0.036 | 0.849 |
| Lesson type (SG) | 0.132 | 0.096 | 2 | 4.517 | 0.105 |
| Lesson type (EG) | 0.187 | 0.090 |  |  |  |
| Year | -0.019 | 0.077 | 1 | 0.081 | 0.776 |
| Course | 0.010 | 0.084 | 1 | 0.014 | 0.905 |
| Application-directed learning score | 0.006 | 0.008 | 1 | 0.607 | 0.436 |
| Student | 0.183 |  |  |  |  |
| Student\|Lesson type (SG) | 0.000 |  |  |  |  |
| Student\|Lesson type (EG) | 0.000 |  |  |  |  |
| Subject | 0.000 |  |  |  |  |
|  |  |  |  |  |  |
| Year 2015 | | | | | |
| (Intercept) | -0.306 | 0.605 |  |  |  |
| BeforeAfter*Lesson type (SG) | -0.123 | 0.185 | 2 | 0.465 | 0.792 |
| BeforeAfter*Lesson type (EG) | -0.090 | 0.179 |  |  |  |
| BeforeAfter | 0.107 | 0.136 | 1 | 0.193 | 0.661 |
| Lesson type (SG) | 0.218 | 0.129 | 2 | 4.308 | 0.116 |
| Lesson type (EG) | 0.223 | 0.126 |  |  |  |
| Course | 0.061 | 0.137 | 1 | 0.199 | 0.655 |
| Application-directed learning score | 0.002 | 0.011 | 1 | 0.043 | 0.835 |
| Student | 0.225 |  |  |  |  |
| Student\|Lesson type (SG) | 0.000 |  |  |  |  |
| Student\|Lesson type (EG) | 0.000 |  |  |  |  |
| Subject | 0.000 |  |  |  |  |
|  |  |  |  |  |  |
| Year 2016 | | | | | |
| (Intercept) | -0.877 | 0.471 |  |  |  |
| BeforeAfterLT (Before)*Lesson type (EG) | -0.133 | 0.188 | 4 | 0.854 | 0.931 |
| BeforeAfterLT (Before)*Lesson type (SG) | -0.102 | 0.216 |  |  |  |
| BeforeAfterLT (LT)*Lesson type (EG) | -0.099 | 0.185 |  |  |  |
| BeforeAfterLT (LT)*Lesson type (SG) | 0.024 | 0.220 |  |  |  |
| BeforeAfterLT (Before) | 0.055 | 0.131 | 2 | 0.213 | 0.899 |
| BeforeAfterLT (LT) | 0.055 | 0.130 |  |  |  |
| Lesson type (SG) | 0.005 | 0.148 | 2 | 2.345 | 0.310 |
| Lesson type (EG) | 0.175 | 0.131 |  |  |  |
| Course | -0.039 | 0.085 | 1 | 0.254 | 0.615 |
| Application-directed learning score | 0.022 | 0.082 | 1 | 3.333 | 0.068 |
| Student | 0.091 |  |  |  |  |
| Student\|Lesson type (SG) | 0.000 |  |  |  |  |
| Student\|Lesson type (EG) | 0.000 |  |  |  |  |
| Subject | 0.000 |  |  |  |  |
